# Advanced Hydrogel Systems for Local Anesthetic Delivery: Toward Prolonged and Targeted Pain Relief

**DOI:** 10.3390/gels11020131

**Published:** 2025-02-12

**Authors:** Jin-Oh Jeong, Minjoo Kim, Seonwook Kim, Kyung Kwan Lee, Hoon Choi

**Affiliations:** 1 Wake Forest Institute for Regenerative Medicine (WFIRM), Wake Forest School of Medicine, Winston-Salem, NC 27157, USA; jijeong@wakehealth.edu (J.-O.J.); kylee@wakehealth.edu (K.K.L.); 2 Department of Anesthesiology and Pain Medicine, Seoul St. Mary’s Hospital, College of Medicine, The Catholic University of Korea, Seoul 06591, Republic of Korea; minju1025@catholic.ac.kr; 3 Department of Internal Medicine, Wake Forest School of Medicine, Winston-Salem, NC 27157, USA; seokim@wakehealth.edu

**Keywords:** local anesthetics, hydrogels, pain management, stimuli-responsive materials, 3D printing, tissue engineering

## Abstract

Local anesthetics (LAs) have been indispensable in clinical pain management, yet their limitations, such as short duration of action and systemic toxicity, necessitate improved delivery strategies. Hydrogels, with their biocompatibility, tunable properties, and ability to modulate drug release, have been extensively explored as platforms for enhancing LA efficacy and safety. This narrative review explores the historical development of LAs, their physicochemical properties, and clinical applications, providing a foundation for understanding the integration of hydrogels in anesthetic delivery. Advances in thermoresponsive, stimuli-responsive, and multifunctional hydrogels have demonstrated significant potential in prolonging analgesia and reducing systemic exposure in preclinical studies, while early clinical findings highlight the feasibility of thermoresponsive hydrogel formulations. Despite these advancements, challenges such as burst release, mechanical instability, and regulatory considerations remain critical barriers to clinical translation. Emerging innovations, including nanocomposite hydrogels, biofunctionalized matrices, and smart materials, offer potential solutions to these limitations. Future research should focus on optimizing hydrogel formulations, expanding clinical validation, and integrating advanced fabrication technologies such as 3D printing and artificial intelligence-driven design to enhance personalized pain management. By bridging materials science and anesthetic pharmacology, this review provides a comprehensive perspective on current trends and future directions in hydrogel-based LA delivery systems.

## 1. Introduction

Effective pain management is a cornerstone of modern medicine, essential not only for alleviating patient discomfort but also for improving clinical outcomes across surgical, medical, and chronic care settings [1]. Pain, a complex multidimensional experience, profoundly impacts physical, emotional, and psychological well-being [2]. Uncontrolled pain exacerbates stress responses, delays recovery, and diminishes quality of life, highlighting the urgent need for reliable and effective analgesic solutions [3]. Among the various modalities for pain control, local anesthetics (LAs) are indispensable due to their ability to provide targeted, reversible nerve blockade without inducing systemic sedation [4].

Despite their widespread use and efficacy, conventional LAs face significant limitations, including short duration of action, frequent re-administration requirements, and the risk of systemic toxicity due to non-specific drug diffusion [5]. For instance, commonly used amide-based agents like bupivacaine and lidocaine typically provide effective durations ranging from one to several hours, depending on dosage and administration technique [6]. In the perioperative setting, the limited duration of action of bupivacaine often necessitates continuous infusion or multiple bolus doses to maintain effective pain relief, increasing the risk of catheter-associated complications [7]. Similarly, in dental procedures, lidocaine’s short activity window frequently requires additional injections during extended treatments, causing patient discomfort and heightened risk of systemic toxicity, such as serious cardiac and neurological complications at high plasma concentrations [8]. Additionally, non-specific diffusion often reduces therapeutic efficacy and leads to unintended effects in off-target tissues [9]. These challenges necessitate innovative approaches to improve LA delivery and performance.

To overcome these limitations, various advanced drug delivery systems have been developed, including liposomal formulations, polymeric microspheres, and hydrogel-based carriers [10]. Among these, liposomal encapsulation and polymeric microspheres have been extensively studied as sustained-release platforms for LAs [11]. Exparel (Pacira BioSciences, Inc., Parsippany, NJ, USA), a liposomal bupivacaine formulation, remains the only FDA-approved nanosystem for prolonged analgesia, demonstrating extended pain relief of up to 72 h [12]. Similarly, polymeric microspheres have been investigated for controlled drug release, utilizing gradual polymer degradation to maintain analgesic effects over extended periods [13].

While these technologies have shown efficacy in prolonging LA duration and reducing systemic toxicity, they are associated with several challenges, including burst release, formulation instability, and high production costs [10]. Furthermore, achieving precise localization at the target site remains a challenge, often leading to unpredictable drug diffusion [11,13]. Given these limitations, hydrogels have emerged as a complementary approach, offering a tunable, biocompatible, and localized drug delivery system with distinct advantages over particulate-based formulations [14,15,16].

Hydrogels have been extensively utilized in pharmaceutical and biomedical applications, including wound healing, tissue engineering, and controlled drug delivery [16]. In the context of LA delivery, hydrogels represent a versatile platform rather than an entirely novel approach. Their application in sustained LA release has gained increasing interest as an alternative to conventional carrier systems such as liposomal encapsulation and polymeric microspheres [10]. Unlike liposomal and microsphere-based formulations, which primarily function as particulate carriers, hydrogels form a localized depot that can adhere to tissues, providing sustained and site-specific drug release [14,15]. This localized retention can be particularly advantageous for perineural administration, intra-articular injections, and wound infiltration, where prolonged analgesia at the target site is critical [17].

Furthermore, stimuli-responsive hydrogels, which modulate drug release in response to physiological conditions such as temperature, pH, or enzymatic activity, provide greater precision in drug delivery compared to traditional sustained-release carriers [18]. Recent advancements in hydrogel technology, including biofunctionalization, nanocomposite integration, and enhanced injectability, have further expanded their clinical applicability, making them suitable for precision medicine approaches in perioperative and chronic pain management [19].

Beyond extending analgesic duration, hydrogel-based systems enable combination therapies, incorporating LAs with anti-inflammatory agents, regenerative molecules, or growth factors [20,21,22]. This multi-functional approach not only enhances pain relief but also facilitates tissue recovery, aligning with modern pain management strategies [16]. Furthermore, hydrogel-based formulations can be designed for various delivery routes, including perineural injections, intra-articular administration, transdermal patches, and implantable hydrogel depots, broadening their potential use in acute and chronic pain management [5,14,16,23].

This review examines the development and potential of hydrogel-based systems for LA delivery, specifically emphasizing their ability to address challenges associated with conventional formulations while introducing novel functionalities such as stimuli-responsive release and combination therapies. By focusing on the integration of hydrogels with advanced materials and precision medicine approaches, this review provides unique insights into future directions for transforming acute and chronic pain management. It begins by exploring the historical evolution of LAs and their delivery systems, from natural agents like cocaine to sophisticated synthetic formulations. This is followed by a detailed discussion of the properties of LAs, including their classification, molecular structure, pharmacokinetics, and clinical applications. Subsequently, the properties and mechanisms of hydrogels are emphasized, highlighting their compatibility with LAs and their role in overcoming challenges associated with conventional formulations. Preclinical and clinical studies showcasing the efficacy and safety of hydrogel-LA systems are critically reviewed, providing evidence of their transformative potential in both acute and chronic pain management.

**Figure 1:**
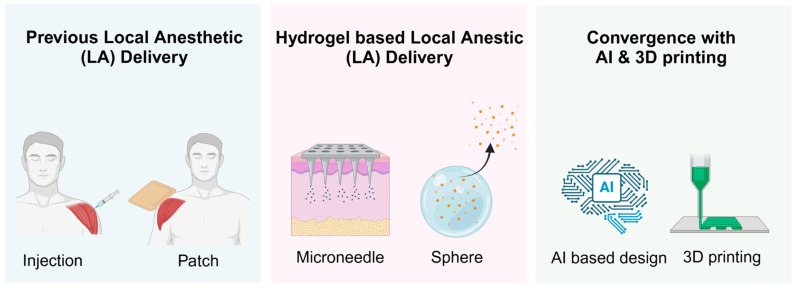
Hydrogel Systems for Local Anesthetic Delivery.

Looking ahead, combining hydrogels with advanced technologies, including stimuli-responsive materials and personalized designs facilitated by 3D printing, offers significant potential to transform pain management approaches. By synthesizing insights from materials science, pharmacology, and clinical anesthesiology, this review highlights advancements and future directions in hydrogel-LA systems, aiming to inspire further innovation and collaboration in this multidisciplinary field (Figure 1).

## 2. Historical Perspective

### 2.1. Early Use of Natural Local Anesthetics

The history of LAs spans centuries, with natural substances being utilized for pain relief long before the advent of modern medicine. Among these, cocaine, derived from the coca plant (*Erythroxylon coca*), holds a pivotal role as the first clinically effective LA. Indigenous South American populations chewed coca leaves for their stimulant and mild analgesic properties for centuries, embedding the plant in cultural and ritualistic practices [24]. Cocaine was first isolated as a pure chemical substance by Albert Niemann in 1860, who identified and named the alkaloid “cocaine” during his doctoral research. However, its anesthetic effects were first recognized in 1884 by Carl Koller, an Austrian ophthalmologist, who demonstrated its utility as a topical anesthetic during ophthalmic surgery [25]. This breakthrough showcased the potential for localized anesthesia without systemic sedation, revolutionizing pain management by enabling safer and more targeted surgical interventions.

Despite its transformative role, cocaine’s clinical application was limited by significant drawbacks, including high toxicity at therapeutic doses, addictive potential, and a short duration of action. Furthermore, its narrow therapeutic window increased the risk of severe cardiovascular and neurological complications in overdose scenarios [26]. These limitations catalyzed the search for alternative compounds, ultimately paving the way for the development of synthetic LAs.

### 2.2. Development of Synthetic Local Anesthetics

The quest for safer and more effective LAs began in earnest with the development of procaine, the first synthetic ester-based anesthetic, introduced in 1905 by German chemist Alfred Einhorn. Marketed under the trade name “Novocaine”, procaine addressed many of cocaine’s limitations, such as its high systemic toxicity and addictive potential [27]. However, procaine’s clinical utility was constrained by its relatively short duration of action and slow onset, making it less suitable for lengthy surgical procedures [28]. This drove the need for anesthetics with improved pharmacological profiles.

A significant leap forward occurred with the advent of amide-based LAs. Lidocaine, developed in 1943 by Swedish chemist Nils Löfgren, became the first amide anesthetic and remains a cornerstone of clinical anesthesia to this day [29]. Compared to procaine, lidocaine exhibited a faster onset, longer duration, and greater chemical stability, enabling its use across diverse medical applications ranging from minor surgical procedures to complex regional anesthesia techniques [28].

The development of synthetic LAs was initially driven by trial and error rather than a precise understanding of the physiological mechanisms underlying natural anesthetics like cocaine [30]. However, as knowledge of ion channel function and nerve conduction improved, rational drug design played an increasing role in optimizing anesthetic properties for safety and efficacy [31].

Further advancements led to the development of tetracaine, a long-acting ester anesthetic widely used in spinal and ophthalmic anesthesia, and prilocaine, an amide anesthetic known for its reduced toxicity and application in regional anesthesia. These were followed by bupivacaine, renowned for its prolonged action, and ropivacaine, which maintained bupivacaine’s efficacy while significantly reducing cardiotoxicity [32]. These innovations marked a critical evolution in locoregional anesthesia, addressing safety concerns while expanding clinical applications such as epidural analgesia during childbirth, nerve blocks for postoperative pain, and infiltration anesthesia in outpatient settings [27]. Table 1 summarizes the key characteristics, uses, and limitations of natural and synthetic LAs. This progression from ester- to amide-based anesthetics underscored the importance of continuous innovation to meet evolving clinical demands.

### 2.3. Evolution of Local Anesthetic Delivery Systems

Advancements in LA chemistry were paralleled by progress in delivery methods, with the goal of improving both the efficacy and safety of these drugs. Initially, bolus injections were the primary mode of administration. While effective for short-term pain relief, frequent dosing was often necessary to maintain analgesia, increasing the risk of cumulative toxicity and patient discomfort [33].

To overcome these challenges, technologies such as continuous infusion pumps and perineural catheters were introduced. These systems facilitated steady drug delivery, reducing intervention frequency and improving pain control consistency. Epidural catheters became a cornerstone of labor analgesia, while continuous nerve block infusions gained prominence in postoperative pain management for major surgical procedures [34,35]. However, these methods were not without challenges, including the need for specialized equipment, meticulous monitoring, and risks such as infection, catheter dislodgement, and systemic toxicity [36].

The development of liposomal formulations marked a significant advancement in LA delivery. Liposomal bupivacaine (Exparel) encapsulates the drug within lipid bilayers, allowing controlled release over extended periods, up to 72 h [12]. This innovation reduced the need for repeated dosing and improved patient compliance [37]. Nonetheless, limitations such as inconsistent release profiles, high production costs, and limited stability have prompted ongoing exploration of alternative delivery platforms [38,39]. Table 2 summarizes the key delivery methods, highlighting their advantages, limitations, and clinical applications.

### 2.4. Emergence of Hydrogels in Local Anesthetic Delivery

Hydrogels, three-dimensional networks of crosslinked polymers capable of retaining significant amounts of water, have been explored for various biomedical applications, including drug delivery, wound healing, and tissue engineering [15]. Their application in LA delivery has gained increased attention in recent years, as researchers investigate their potential for sustained and localized drug release [16].

By encapsulating LAs within hydrogel matrices, these materials confine drug release to the target site, minimizing systemic exposure and associated toxicity risks [40,41]. Furthermore, the controlled release mechanisms prevent rapid plasma concentration spikes, reducing adverse effects associated with bolus injections or particulate-based carriers [15]. The structural versatility of hydrogels allows for customization to meet specific clinical needs, making them a transformative tool in pain management [23]. These attributes not only improve the safety profile of LAs but also reduce administration frequency, enhancing overall treatment efficacy and patient convenience.

### 2.5. Toward the Integration of Multifunctional Systems

The integration of multifunctional delivery systems represents the next frontier in LA development. Modern hydrogels are being engineered to combine pain relief with additional therapeutic benefits, such as anti-inflammatory effects or tissue regeneration [42,43]. For instance, hydrogels co-loaded with LAs and anti-inflammatory agents can simultaneously address pain and inflammation, while those incorporating growth factors show promise for facilitating postoperative tissue repair [44].

Emerging technologies, such as stimuli-responsive hydrogels, further illustrate the potential of these systems. These materials regulate drug release according to external factors like temperature or pH fluctuations, aligning with the principles of precision medicine [45,46]. As research progresses, these innovative platforms are anticipated to become integral to personalized pain management, offering tailored solutions for individual patient needs [47].

The evolution of LAs agents and their delivery systems underscores the field’s commitment to addressing clinical challenges while enhancing patient outcomes. Future advancements in hydrogel-based LA delivery are expected to focus on optimizing drug release kinetics, improving biocompatibility, and integrating patient-specific formulations to enhance efficacy and safety in clinical applications.

## 3. Properties of Local Anesthetics

### 3.1. Classification of Local Anesthetics

LAs are broadly categorized into two primary classes: ester-based and amide-based, distinguished by the chemical structure of their intermediate chain. This structural difference significantly influences their metabolic pathways, duration of action, and adverse effect profiles [48].

Ester-based anesthetics, such as procaine and tetracaine, were among the earliest synthetic LAs used in clinical practice. These agents are metabolized primarily by plasma cholinesterase, which contributes to their shorter duration of action [49]. While rapid metabolism reduces systemic accumulation, it necessitates more frequent dosing to maintain analgesia. A notable limitation is the increased risk of allergic reactions due to the production of para-aminobenzoic acid as a metabolic byproduct [8]. For example, procaine is associated with limited efficacy in prolonged procedures due to its rapid hydrolysis and susceptibility to allergic responses, reducing its clinical utility in favor of more stable alternatives [50].

In contrast, amide-based anesthetics, including lidocaine, bupivacaine, and ropivacaine, have largely replaced ester-based compounds due to their superior safety and efficacy profiles. These agents are metabolized in the liver via cytochrome P450 enzymes, which confer greater chemical stability and longer duration of action [51]. For instance, bupivacaine offers a prolonged duration suitable for epidural or spinal anesthesia, though its higher lipid solubility increases the risk of cardiotoxicity at elevated plasma concentrations [52]. Ropivacaine, developed to address this limitation, retains the prolonged action of bupivacaine while significantly reduced cardiotoxic potential, making it a preferred choice in regional and continuous infusion techniques [53]. Additionally, amide-based anesthetics exhibit a lower incidence of hypersensitivity reactions, further broadening their clinical applications [8].

The shift from ester-based to amide-based anesthetics reflects a pivotal milestone in the history of anesthetic development, highlighting the pursuit of agents that optimize safety, efficacy, and convenience [32]. While ester-based LAs remain useful for specific scenarios, amide-based agents dominate contemporary practice due to their superior pharmacokinetic and pharmacodynamic properties [54].

### 3.2. Molecular Structure and Mechanism of Action

The molecular structure of LAs consists of three essential components: a lipophilic aromatic ring, an intermediate ester or amide chain, and a hydrophilic amine group. The lipophilic aromatic ring facilitates membrane penetration, allowing the drug to reach its site of action. The intermediate chain influences metabolic stability and clearance, while the hydrophilic amine group ensures solubility and interaction with cellular components [55].

Once inside the neuron, LAs bind to voltage-gated sodium channels in their inactivated state, blocking sodium ion influx and preventing action potential initiation and propagation. This blockade results in a reversible nerve conduction block. Key physicochemical properties, including lipophilicity, pKa, and protein binding affinity, determine the onset, potency, and duration of action [31]. For example, bupivacaine’s high lipid solubility accounts for its prolonged action despite a slower onset [56].

### 3.3. Pharmacokinetics and Pharmacodynamics

The pharmacokinetics of LAs, encompassing absorption, distribution, metabolism, and excretion, are influenced by both their physicochemical properties and the route of administration [48]. In highly vascularized areas, such as the intercostal space, rapid absorption increases the risk of systemic toxicity [36]. To mitigate this risk, vasoconstrictors like epinephrine are often co-administered to prolong local drug effects and reduce systemic absorption [57]. Systemic clearance of LAs is largely determined by their protein binding affinity and lipid solubility, which influence both their distribution and elimination. For example, bupivacaine and ropivacaine exhibit high protein binding, at approximately 95% and 94%, respectively, which limits their free drug concentration in circulation and extends their half-life [54]. Consequently, bupivacaine has an elimination half-life of approximately 2.7 h, while ropivacaine has a slightly longer half-life of around 4.2 h, contributing to their prolonged duration of action compared to less protein-bound agents like lidocaine [51].

Pharmacodynamically, LAs produce a differential blockade, with smaller, myelinated fibers responsible for pain and temperature sensation being more susceptible than larger motor fibers [49]. This sequence of blockade—sympathetic fibers → pain and temperature → touch and proprioception → motor function—enables selective sensory blockade in clinical applications [48]. The duration of anesthesia also varies significantly among LAs, with bupivacaine providing extended analgesia for up to 6–8 h, whereas lidocaine typically lasts 1.5–2 h, depending on the formulation and site of administration [6]. These pharmacokinetic and pharmacodynamic properties are particularly relevant in hydrogel-based drug delivery systems, where controlled and sustained release can be leveraged to enhance anesthetic efficacy while minimizing systemic exposure and toxicity.

### 3.4. Clinical Applications and Limitations

LAs are indispensable in modern medical practice, providing targeted analgesia for various clinical applications. Lidocaine, a versatile LA, is frequently employed in infiltration anesthesia, peripheral nerve blocks, and intravenous regional anesthesia. The rapid onset and intermediate duration of lidocaine make it ideal for short procedures [58]. Bupivacaine, known for its prolonged duration, is favored for epidural and spinal anesthesia as well as postoperative pain management via peripheral nerve blocks [6]. Ropivacaine, with reduced cardiotoxicity compared to bupivacaine, has become a popular choice for regional anesthesia and continuous infusions [59].

Despite their utility, LAs are not without limitations. A critical concern is LA systemic toxicity (LAST), a life-threatening condition caused by excessive plasma drug levels, often due to accidental intravascular injection [9]. Symptoms of LAST include central nervous system excitation (e.g., agitation, seizures) followed by cardiovascular depression (e.g., bradycardia, hypotension) [8]. Lipid emulsion therapy, which acts as a drug scavenger, is the primary treatment for LAST [60].

Another limitation is the short duration of many LAs, requiring repeated administration or continuous infusion, which increases logistical challenges and cumulative toxicity risks [61]. Additionally, LAs are less effective in inflamed or infected tissues due to an acidic environment that reduces the proportion of non-ionized drug molecules capable of membrane penetration [48].

### 3.5. Emerging Trends in Local Anesthetic Development

Emerging trends in LA research seek to address systemic toxicity, improve duration, and enhance specificity [10]. Novel agents, such as site-1 sodium channel blockers and peptide-based anesthetics, offer the potential for prolonged and selective nerve blockade with improved safety profiles [62,63]. These agents differ mechanistically from conventional LAs by targeting distinct molecular pathways, reducing off-target effects [64]. For instance, site-1 sodium channel blockers inhibit sodium influx at alternate sites, minimizing toxicity [65]. Similarly, peptide-based anesthetics use bioengineered structures to enhance selectivity, making them ideal for high-risk populations [62].

Advanced drug delivery systems, including widely explored carriers such as hydrogels, liposomal formulations, and polymeric microspheres, have played a crucial role in optimizing LA administration by enabling prolonged, localized release and minimizing systemic exposure [11,13,15]. Injectable hydrogels, for example, provide extended release at target sites, reducing the need for frequent dosing [15]. Liposomal formulations of bupivacaine have also demonstrated clinical success in extending analgesic duration [37]. The convergence of novel agents and delivery systems underscores their combined potential to refine the fundamental properties of LAs, addressing key pharmacokinetic and pharmacodynamic challenges (Table 3). These advancements represent a transformative step toward safer, more effective, and increasingly personalized approaches to locoregional anesthesia.

## 4. Introduction to Hydrogels

### 4.1. Definition and Basic Characteristics

Hydrogels, defined as three-dimensional networks of hydrophilic polymers capable of absorbing and retaining substantial amounts of water, provide unique advantages for drug delivery applications. The substantial water-retaining capacity of these materials, coupled with structural versatility and inherent biocompatibility, enables accurate control of drug release dynamics and enhances therapeutic outcomes [14]. Additionally, their structural similarity to the extracellular matrix (ECM), along with their tunable properties, has driven widespread adoption in biomedical applications [18].

Hydrogels are formed by polymer chains crosslinked through physical or chemical interactions, which creates cohesive three-dimensional networks [16]. Their hydrophilic nature allows for significant water absorption, often exceeding 90% of their total weight. This imparts softness, flexibility, and permeability, which closely replicate the characteristics of natural tissues [66]. Furthermore, their biocompatibility reduces immune responses, enhancing their suitability for medical applications [67].

The mechanical and functional properties of hydrogels can be tailored by modifying factors such as polymer composition, crosslinking density, and environmental conditions [68]. For instance, increasing crosslinking density enhances mechanical stability but may reduce swelling capacity and drug-loading potential [45]. Hydrogels can also be engineered to respond to specific environmental stimuli, such as pH changes, temperature variations, or enzymatic activity, enabling controlled and sustained drug release [69,70,71].

Another notable advantage of hydrogels is their capacity to replicate the ECM, offering both structural and functional support for cellular proliferation and differentiation [72,73]. This characteristic renders them exceptionally well-suited for use in tissue engineering and regenerative medicine. Furthermore, hydrogels exhibit tunable degradation rates, ranging from rapid breakdown for short-term drug delivery to prolonged stability for sustained applications [74,75]. These attributes underscore their versatility in diverse medical applications.

### 4.2. Classification of Hydrogels

Hydrogels are categorized based on their origin, crosslinking method, responsiveness to stimuli, and chemical composition [76]. Each classification offers unique benefits tailored to specific applications.

Natural hydrogels, including those based on alginate, chitosan, and hyaluronic acid, are sourced from biological origins, which endows them with inherent biocompatibility and biodegradability. These materials closely mimic natural tissue environments, making them ideal for wound healing and drug delivery [77]. However, compared to synthetic hydrogels, they may exhibit limitations such as reduced mechanical strength and variability in composition, which can hinder reproducibility and large-scale application [78]. In contrast, synthetic hydrogels, including those based on polyethylene glycol and polyvinyl alcohol, provide enhanced control over mechanical and chemical properties, enabling precise customization for biomedical needs [79]. Synthetic hydrogels also demonstrate superior reproducibility and stability, making them more suitable for large-scale applications [73,80]. However, their lack of inherent bioactivity may require additional modifications to promote cell interactions and biodegradability [81].

Crosslinking methods further classify hydrogels. Physically crosslinked hydrogels rely on reversible interactions, including hydrogen bonding, ionic interactions, or hydrophobic forces [82]. These hydrogels are often biodegradable and can adapt to environmental changes. In contrast, chemically crosslinked hydrogels form covalent bonds, offering enhanced mechanical strength and prolonged stability, which are essential for long-term applications [83].

Smart hydrogels are advanced materials that change their physical or chemical properties in response to external stimuli [84]. For example, thermoresponsive hydrogels undergo sol-gel transitions near physiological temperatures, allowing less invasive delivery methods and targeted drug release [74]. pH-sensitive hydrogels expand or contract based on pH changes, facilitating localized drug delivery within acidic conditions, including inflamed tissues or tumor regions [45,75,85]. Other stimuli-responsive hydrogels react to light, redox conditions, or biomolecules, broadening their applicability in precision medicine [86].

Hydrogel composition significantly influences their properties. Homopolymer hydrogels, composed of a single polymer type, offer design simplicity and uniformity. Copolymer hydrogels are formed by combining two or more monomers, allowing for the tailoring optimization of properties such as improved mechanical strength or increased drug-loading capacity [14]. Interpenetrating polymer network hydrogels, integrating multiple crosslinked networks, provide superior mechanical and functional versatility, making them suitable for applications requiring robustness under stress or varying conditions [87,88].

### 4.3. Mechanisms of Drug Delivery

**Figure 2:**
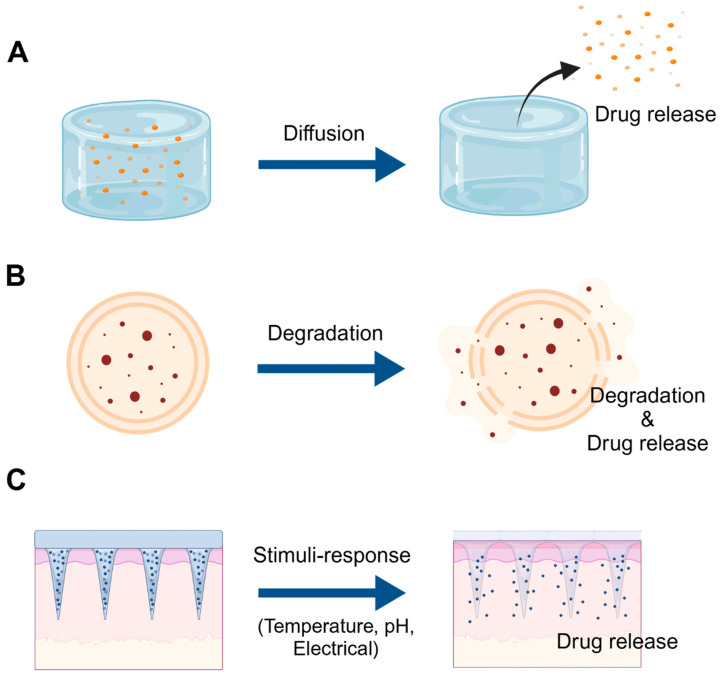
Key Mechanisms of Drug Release from Hydrogels: (**A**) Diffusion-controlled, (**B**) Degradation-controlled, (**C**) Stimuli-response.

The mechanisms underlying drug release from hydrogels are closely tied to their structural and physicochemical properties [15]. Three primary mechanisms facilitate controlled and sustained drug delivery (Figure 2).

The first mechanism, diffusion-controlled release, depends on the passive diffusion of drug molecules from the hydrogel matrix into the surrounding medium [89]. Factors such as hydrogel mesh size, drug molecular weight, and interactions between the drug and hydrogel material determine the release rate [90,91]. This mechanism is effective for small-molecule drugs, with sustained release achievable by adjusting the hydrogel’s structural parameter [92].

The second mechanism is degradation-controlled release, in which the hydrogel matrix gradually disintegrates, allowing the encapsulated drugs to be released over time [93]. This approach is advantageous for site-specific and sustained delivery, as degradation rates can be tailored through chemical composition and crosslinking density modifications [41]. Biodegradable hydrogels are frequently employed in tissue engineering and post-surgical applications, as they gradually degrade after achieving their therapeutic function [74].

The third mechanism, stimuli-responsive release, utilizes the hydrogel’s capacity for structural or chemical transformations triggered by external stimuli [84]. For instance, hydrogels responsive to pH changes selectively release drugs under low-pH conditions, including inflamed tissues or tumor sites [45,75,85]. Thermoresponsive hydrogels align drug release with localized temperature changes. These smart hydrogels enable precision medicine by providing on-demand drug release tailored to clinical scenarios [86,94].

The versatility of hydrogels for therapeutic applications arises from biocompatibility, tunable characteristics, and responsiveness to external stimuli. These features specifically enhance the delivery of LAs by enabling prolonged and targeted pain relief, reducing systemic side effects, and improving patient outcomes in clinical practice. This combination positions hydrogels as transformative platforms for controlled therapeutic delivery, particularly for LAs, where sustained and targeted pain relief is critical [85,87]. The key features and applications of these mechanisms are summarized in Table 4 below.

## 5. Hydrogels for Local Anesthetic Delivery

### 5.1. Hydrogels as Drug Carriers for Local Anesthetics

The integration of hydrogels with LAs marks a significant advancement in pain management, addressing limitations such as short drug duration, systemic toxicity, and inconsistent release profiles [10]. Hydrogels function as adaptable carriers, encapsulating LAs to enable prolonged and targeted drug delivery while minimizing side effects and enhancing efficacy across diverse physiological environments [15]. This synergy is especially beneficial in perioperative and postoperative contexts, where prolonged analgesia reduces opioid dependence and enhances recovery [40,69,72].

One primary advantage of hydrogels lies in their ability to stabilize LAs, preventing rapid degradation or diffusion at the site of administration [14]. Thermoresponsive hydrogels exhibit a sol-to-gel transition at physiological temperatures, allowing injectable formulations to form a depot at the target site, leading to prolonged drug retention and controlled release [90]. These systems are widely used in oncology for delivering chemotherapeutic agents like doxorubicin, where the phase transition helps localize the drug and minimize systemic toxicity [95]. Clinical and preclinical studies underscore these benefits, with formulations incorporating ropivacaine or lidocaine demonstrating extended analgesic effects and reduced systemic exposure compared to traditional methods [41,96].

Controlled release mechanisms further enhance the utility of hydrogels for LA delivery. Diffusion-based release, degradation-controlled release, and stimuli-responsive release ensure sustained and localized analgesic effects [97]. Stimuli-responsive hydrogels adjust drug release in response to environmental factors such as pH, temperature, or enzymatic activity, making them highly adaptable for dynamic physiological conditions [84]. For instance, pH-sensitive hydrogels have been extensively used in oral drug delivery systems for peptides and proteins, where they protect the drug from gastric degradation and release it in the intestines [98]. This adaptability improves therapeutic outcomes by tailoring drug release to local physiological conditions [92,99].

Beyond controlled release, multifunctional hydrogels are being developed to provide additional therapeutic benefits, such as anti-inflammatory effects or tissue regeneration [16]. These hydrogels can be functionalized with bioactive molecules, such as anti-inflammatory drugs, growth factors, or antimicrobial agents, to enhance postoperative healing while providing sustained analgesia [20,21,22]. In wound healing applications, hydrogels incorporating silver nanoparticles have demonstrated both antimicrobial protection and controlled antibiotic release, reducing infection risks while supporting tissue regeneration [100]. By integrating multiple functionalities within a single platform, these hydrogels align with modern pain management strategies that emphasize both symptom control and tissue recovery.

The physicochemical properties of LAs can also be augmented through hydrogel engineering. Hybrid systems incorporating nanoparticles, liposomes, or polymeric micelles within hydrogel matrices have demonstrated dual benefits of targeted delivery and sustained release [101]. For example, lipid-based nanocarrier-hydrogel hybrids have shown enhanced bioavailability in complex anatomical sites, such as surgical wounds or joint spaces [45,69,74].

Incorporating biocompatible and biodegradable polymers into hydrogels ensures minimal immune response while facilitating degradation into non-toxic byproducts. This characteristic is crucial for chronic pain management and postoperative care [102]. Advanced nanocomposite hydrogels, incorporating materials like graphene oxide or chitosan derivatives, further enhance mechanical strength and drug retention, making them promising candidates for clinical applications [87,96,103].

### 5.2. Preclinical Evidence: Bridging Concept and Practice

Preclinical investigations into hydrogel-based LA delivery systems highlight their transformative potential. These innovative systems address critical limitations of conventional LAs, such as short duration of action, burst release profiles, and systemic toxicity, by leveraging breakthroughs in materials science and bioengineering [10]. Hydrogels have demonstrated exceptional promise as biocompatible and customizable platforms for achieving prolonged and targeted analgesia [16]. Various types of hydrogels have been explored for their ability to enhance drug stability, control release kinetics, and provide additional therapeutic benefits. A summary of recent preclinical advancements in hydrogel-based LA delivery systems is provided in Table 5.

Among these, thermoresponsive hydrogels have been widely studied for their ability to undergo sol-to-gel transitions at physiological temperatures, forming an in situ depot that enables sustained drug release through a diffusion-controlled mechanism [108]. These materials leverage phase transition properties to enhance drug retention and prolong LA effects. Poly(N-isopropylacrylamide) (PNIPAM), a thermoresponsive polymer with a lower critical solution temperature (LCST, ~32 °C), has been extensively explored in these systems. In a sciatic nerve block model, anionically functionalized PNIPAM nanogels loaded with bupivacaine extended sensory blockade to approximately 9 h, nearly doubling the duration achieved with conventional formulations [104]. Similarly, Pluronic F127, a triblock copolymer of poly(ethylene oxide)-poly(propylene oxide)-poly(ethylene oxide), has been investigated for its ability to improve anesthetic stability. In a canine model, a bupivacaine-loaded Pluronic F127 hydrogel significantly extended sensory blockade to 8.0 ± 1.6 h and motor blockade to 9.3 ± 1.6 h, compared to 3.7 ± 2.0 and 4.6 ± 1.9 h with standard bupivacaine injections [71]. Poloxamer-based thermoresponsive hydrogels, primarily composed of Poloxamer 407 (poly(ethylene oxide)-poly(propylene oxide)-poly(ethylene oxide)), have also shown promise for localized pain control and tissue repair. In a wound healing model, lidocaine hydrochloride-loaded poloxamer hydrogels exhibited rapid gelation within 1–3 min and prolonged anesthetic effects, with clinical trials demonstrating anesthesia onset at 46.5 ± 22.5 s and maintenance for 202.5 ± 41.0 s [41]. Another thermoresponsive formulation, poly(N-isopropylacrylamide-co-dimethylbutyrolactone acrylamide-co-Jeffamine M-1000 acrylamide) (PNDJ), has been evaluated in orthopedic applications. In a rabbit knee surgery model, intraarticular administration of a PNDJ-based hydrogel (SBG004) resulted in sustained analgesia for up to 96 h and systemic bupivacaine release for over 7 d, significantly outperforming liposomal bupivacaine and standard ropivacaine formulations [74].

In addition to thermoresponsive formulations, stimuli-responsive hydrogels modulate drug release based on physiological or environmental triggers through a combination of diffusion-controlled and degradation-controlled mechanisms, enabling precision drug delivery. pH-sensitive hydrogels, commonly formulated using poly(acrylic acid) or methylcellulose, regulate bupivacaine release in response to the acidic microenvironment of inflamed tissues, enabling localized drug action while minimizing systemic exposure [98]. Methylcellulose-based hydrogels exhibited pH-dependent swelling behavior, leading to a sustained and controlled release of bupivacaine, with a 45% cumulative release over 48 h at pH 6.5 compared to only 22% at pH 7.4. These findings underscore their potential for site-specific drug release in inflamed or ischemic tissues [45].

Similarly, enzyme-responsive hydrogels rely on degradation-controlled release, where hydrogel degradation is triggered by upregulated proteases in injured tissues, leading to controlled drug availability. A polydopamine (PDA)-based hybrid hydrogel system demonstrated enzyme-triggered lidocaine release, with degradation rates accelerating in environments rich in matrix metalloproteinases, which are upregulated in inflamed or injured tissues. In vivo studies using a spared nerve injury rat model confirmed that enzyme-mediated lidocaine release from PDA hydrogels significantly prolonged analgesic effects compared to standard lidocaine formulations, highlighting their potential for controlled, on-demand anesthetic delivery [72]. Supramolecular phenolic nanofiller-alginate hydrogels represent another breakthrough in sustained LA delivery. By incorporating phenolic-based nanofillers, these hydrogels extend lidocaine release up to 14 d while maintaining structural integrity, preventing burst release and ensuring a prolonged analgesic effect [92]. Similarly, bacterial cellulose hydrogels have demonstrated the ability to modulate lidocaine release profiles by adjusting network architecture, with hydrogels synthesized using different carbon sources exhibiting controlled drug diffusion properties and sustained anesthetic effects over a 14 d period [105].

Beyond controlled release, multifunctional hydrogels have been designed to integrate additional therapeutic properties while employing multiple drug release mechanisms, including degradation-controlled and stimuli-responsive systems. These systems not only provide sustained analgesia but also contribute to tissue healing, inflammation reduction, and improved functional recovery. A temperature-sensitive multifunctional hydrogel composed of carboxymethyl agarose and N-isopropylacrylamide demonstrated both controlled lidocaine release and accelerated wound healing, with in vivo studies showing a 54.3% reduction in wound bleeding and a 97% improvement in healing rates [106]. Similarly, injectable hydrogel systems combining LAs with adjuvant drugs have shown promise in extending analgesia and improving postoperative outcomes. A ropivacaine-loaded hydrogel incorporating dexmedetomidine exhibited sequential drug release, maintaining sensory and motor blockade for 48 and 36 h, respectively, in a peripheral nerve block model. This dual-drug system provided prolonged pain relief and reduced systemic toxicity compared to conventional ropivacaine formulations [73]. Beyond perioperative pain management, multifunctional hydrogels have been explored for their role in facilitating tissue healing and enhancing functional recovery. A carbon dioxide (CO_2_)-encapsulated Pluronic F127 hydrogel designed for Achilles tendon repair demonstrated continuous bupivacaine release for over 14 d, significantly improving post-surgical mobility and promoting collagen I deposition within the repaired tissue [86]. Furthermore, hydrogels incorporating ropivacaine have been explored for their potential in immunomodulation and tumor recurrence prevention. A ropivacaine-loaded hydrogel co-formulated with a Toll-like receptor 7 agonist significantly enhanced CD8+ T cell infiltration into tumor tissue, reducing postoperative recurrence while maintaining long-lasting analgesia [70]. In another study, a Pluronic F127 hydrogel loaded with cisplatin and ropivacaine (PFCR) was developed to simultaneously manage chemotherapy-induced peripheral neuropathic pain and enhance chemotherapy efficacy. In a tumor-bearing mouse model, PFCR administration significantly prolonged pain relief beyond 10 h and increased CD8+ T cell infiltration into the tumor microenvironment, thereby potentiating the antitumor effects of cisplatin. Mechanistic studies revealed that ropivacaine suppressed tumor cell autophagy, leading to increased major histocompatibility complex class I expression, which in turn improved T cell-mediated immune recognition [75].

To further optimize drug stability and delivery kinetics, recent studies have investigated self-healing hydrogels and pH-modulating hydrogel systems. A sodium deoxycholate-based self-healing hydrogel demonstrated a prolonged ropivacaine release profile, achieving peripheral nerve block for over one week in an inflammatory pain model. The self-healing properties of this hydrogel allowed it to recover structural integrity after mechanical disruption, ensuring consistent drug delivery over time [69]. Similarly, pH-regulating hydrogels have been developed to enhance LA solubility and control release. A biodegradable block copolymer hydrogel loaded with bupivacaine microcrystals and calcium carbonate (CaCO_3_) maintained a stable internal pH, preventing burst release and extending analgesic effects up to 44 h in a sciatic nerve block model [107]. Additionally, hybrid hydrogel systems incorporating nanostructured carriers have been developed to optimize drug delivery. A poloxamer-hyaluronic acid hydrogel was designed to stabilize bupivacaine and ropivacaine, modulating viscosity and micellar interactions to achieve a more controlled release profile [80]. Similarly, gelatin-based hydrogels crosslinked with N-hydroxysuccinimide-polyethylene glycol-N-hydroxysuccinimide have been explored for sciatic nerve block applications, providing a porous three-dimensional structure that enhances drug loading and ensures a slow-release profile, reducing neurotoxicity risks associated with high LA concentrations [40]. Further expanding hydrogel applications in pain management, a lidocaine-nanoparticle-loaded hydrogel coating for chest tubes demonstrated sustained lidocaine release, significantly improving pain tolerance in vivo and offering a novel approach to reducing postoperative discomfort in cardiothoracic surgery patients [103].

Collectively, these findings highlight how engineered hydrogels employ specific drug release mechanisms—diffusion-controlled, degradation-controlled, and stimuli-responsive release—to achieve prolonged analgesia and minimize systemic toxicity. This aligns with the fundamental drug delivery principles discussed in Section 4.3, reinforcing the translational potential of hydrogels in LA applications.

### 5.3. Early Clinical Findings and Applications

The clinical translation of hydrogel-based LA delivery systems represents a significant advancement in pain management strategies, emphasizing prolonged and localized analgesia with reduced systemic side effects [15]. While the body of clinical evidence remains limited, the available studies underscore the transformative potential of these systems across a range of surgical and medical applications. This section synthesizes pivotal clinical studies that demonstrate the transformative potential of hydrogel-based LA delivery systems. Among the different hydrogel formulations, thermoresponsive hydrogels, which facilitate sustained drug release through a diffusion-controlled mechanism, have been the most extensively evaluated in clinical settings, particularly in surgical applications. Additionally, a few studies have investigated standard hydrogel formulations and topical hydrogel-based anesthetic delivery systems in dental and dermatological applications. A comprehensive summary of these studies, categorized by engineering strategies, outlines the hydrogel types, clinical procedures, sample sizes, and key findings that validate their efficacy, safety, and applicability, as provided in Table 6.

Several clinical trials have investigated thermoresponsive hydrogels, which transition from sol to gel at physiological temperatures, forming an in situ depot that minimizes burst release and prolongs drug retention through diffusion-controlled release kinetics. A retrospective study assessed the efficacy of PF72, a thermoresponsive hydrogel composed of Pluronic-based polymers, mixed with 0.75% ropivacaine, in patients undergoing bimaxillary surgery [96]. This study involved 40 participants who underwent LeFort I maxillary osteotomy and sagittal split ramus osteotomy. They were divided into two groups: one group received PF72 directly applied to the surgical site before the procedure, while the other relied solely on intravenous analgesics. Pain intensity was measured at intervals of 3, 6, 24, 48, and 72 h post-surgery using the Numerical Rating Scale (NRS). At 24 h, patients treated with PF72 reported an average NRS score of 4.0 ± 1.3, significantly lower than the 6.4 ± 1.2 in the control group (*p* < 0.05). By 72 h, the scores were 2.6 ± 1.3 for the hydrogel group and 3.4 ± 1.3 for the control group (*p* < 0.05). Additionally, the hydrogel reduced the need for rescue analgesics and was associated with minimal adverse effects, highlighting its potential as a safe and effective alternative for managing postoperative pain in invasive plastic surgery.

A randomized pivotal clinical trial further examined PF72’s application in laparoscopic abdominal surgery [109]. Ninety-nine patients undergoing stomach or colorectal surgery were randomly assigned to receive either 0.75% ropivacaine alone or mixed with PF72. The hydrogel was administered to the subcutaneous fat and muscle at the incision site before surgical closure. Pain levels were assessed over a 72 h period, with the hydrogel group exhibiting significantly lower cumulative NRS scores compared to the control group. Specifically, the area under the curve of NRS scores for wound pain until 72 h was 135.3 ± 49.9 h for the hydrogel group, compared to 188.7 ± 46.1 h for the control group (*p* < 0.001). The frequency of rescue analgesics required in the general ward was comparable between groups (*p* = 0.09), but the hydrogel demonstrated a superior safety profile with no reported adverse events, emphasizing its clinical viability for postoperative pain management.

Poloxamer-based thermoresponsive hydrogels, another promising system, have been clinically evaluated for their ability to enhance anesthetic duration by leveraging diffusion-controlled drug release properties. In a randomized trial involving 61 patients undergoing minimally invasive colorectal surgery, a Poloxamer 407-based hydrogel was compared to a continuous infusion system using On-Q PainBuster device [88]. Patients in the hydrogel group received a single dose of 0.75% ropivacaine-loaded hydrogel directly at the surgical site, whereas the control group relied on continuous infusion of 0.2% ropivacaine over 48 h. The primary endpoint was fentanyl consumption, with secondary endpoints including pain intensity (NRS scores) and hospital stay duration. Both groups achieved comparable pain relief, as measured by fentanyl consumption (*p* = 0.806) and NRS scores (*p* = 0.655). However, patients in the hydrogel group had a significantly shorter average hospital stay of 7.2 ± 1.6 d, compared to 8.4 ± 2.8 d in the infusion group (*p* = 0.045). These findings highlight the hydrogel’s ability to streamline recovery processes, reduce healthcare costs, and maintain effective analgesia without the complexities of infusion systems.

Thoracoscopic pulmonary resection has also benefited from Poloxamer-based thermoresponsive hydrogels. A prospective, randomized trial involving 89 patients compared a poloxamer 407-based ropivacaine hydrogel with a continuous thoracic paravertebral block using the On-Q PainBuster system [110]. Patients in the hydrogel group received a single application of 0.75% ropivacaine hydrogel, while the control group utilized a catheter delivering 0.2% ropivacaine continuously at 4 mg/h for 48 h. The primary outcome measure was total fentanyl consumption, and secondary outcomes included NRS pain scores and frequency of rescue analgesia. Results showed no significant difference in total fentanyl usage (*p* = 0.37). Pain scores were also comparable at all time points up to 72 h postoperatively (*p* > 0.05). However, the hydrogel group benefited from simpler administration and reduced systemic toxicity risks, positioning it as a viable alternative for regional analgesia in thoracic surgeries.

In dental applications, a polyelectrolyte complex-poloxamer hydrogel loaded with lidocaine HCl demonstrated promising results in both preclinical and clinical settings [41]. The hydrogel exhibited rapid gelation within 1–3 min and maintained anesthetic effects for up to 48 h. In clinical trials, the hydrogel provided rapid numbness onset within 46.5 ± 22.5 s and effective anesthesia lasting an average of 202.5 ± 41.0 s. These properties underscore its adaptability for dynamic dental environments, offering prolonged pain relief and enhanced patient satisfaction.

While thermoresponsive hydrogels dominate clinical studies, standard hydrogel formulations have also been explored for needle-free anesthesia. A clinical trial evaluating a liposomal lidocaine-prilocaine hydrogel for palatal anesthesia during upper molar extractions included 40 participants in a randomized, crossover, triple-blinded design [111]. The liposomal formulation achieved a 100% success rate in providing adequate anesthesia, compared to a 40% failure rate in the non-liposomal group (*p* < 0.0001). The average duration of anesthesia was significantly longer in the liposomal group (26.8 ± 7.5 min) than in the non-liposomal group (16.8 ± 4.8 min, *p* < 0.0001). The hydrogel formulation provided effective pain relief without the need for injection, improving patient comfort and procedural outcomes.

Beyond injectable applications, hydrogels have also been studied for topical pain relief. A study assessed a nonaqueous drug-in-matrix system delivering lidocaine for localized pain relief [112]. Clinical evaluations involving 15 healthy volunteers demonstrated enhanced skin permeation and sustained pharmacokinetic activity compared to traditional aqueous patches. The formulation provided effective analgesia over 12 h without significant skin irritation, highlighting its potential for non-invasive pain management across various medical scenarios.

The clinical evaluation of hydrogel-based LA delivery systems remains in its early stages, with thermoresponsive hydrogels being the most extensively studied in clinical trials. These studies demonstrate promising analgesic efficacy, reduced systemic toxicity, and improved postoperative recovery outcomes. Standard and topical hydrogel-based formulations have also shown clinical viability in dental and dermatological applications. By integrating diffusion-controlled, degradation-controlled, and stimuli-responsive mechanisms, these systems optimize LA delivery while minimizing systemic toxicity. However, further large-scale trials are necessary to fully establish their safety, effectiveness, and broader clinical utility.

## 6. Challenges and Innovations in Hydrogel-Local Anesthetic Systems

### 6.1. Optimizing Drug Loading and Sustained Release

Achieving optimal drug loading and controlled release profiles is critical to the success of hydrogel-based LA systems. However, encapsulating high concentrations of LAs without compromising hydrogel structural integrity presents significant challenges [41]. Many formulations experience burst release, a phenomenon where a large portion of the encapsulated drug is released prematurely. This issue undermines the intended prolonged analgesic effects and elevates the risk of systemic toxicity [69]. Furthermore, dynamic physiological environments—characterized by varying pH levels, enzymatic activity, and temperature fluctuations—further complicate consistent and sustained drug release [16].

Recent advances in materials science and bioengineering have provided innovative solutions to these challenges. For instance, advanced crosslinking techniques, such as reversible covalent bonds and supramolecular interactions, have been employed to enhance drug retention and prevent burst release [113]. Nanocarriers, including liposomes, micelles, and polymeric nanoparticles, have been integrated into hydrogel matrices to achieve dual functionality: enhanced drug encapsulation efficiency and controlled, sustained release [114]. These nanostructures provide additional protection for the encapsulated drug, shielding it from premature degradation while facilitating targeted delivery [115].

Emerging computational tools and artificial intelligence (AI) are also transforming hydrogel design [116,117]. These technologies allow researchers to model and predict drug release kinetics under various physiological conditions, enabling the development of hydrogels tailored to specific clinical needs. AI-driven simulations have been instrumental in identifying optimal polymer compositions, crosslinking densities, and release profiles. For example, predictive algorithms can identify ideal formulations that balance prolonged analgesia with minimal systemic exposure, expediting the optimization process [118].

Multilayered hydrogels with gradient structures represent another promising innovation. These systems enable differential release rates, making them suitable for therapeutic regimens requiring sequential or pulsatile drug delivery [119]. By embedding multiple drug reservoirs within distinct layers, these hydrogels can deliver both immediate and sustained effects, catering to complex pain management needs [120].

Stimuli-responsive hydrogels have attracted considerable attention for their capacity to tailor drug release according to local physiological conditions. These systems utilize external stimuli, including changes in pH, temperature, enzymatic activity, or redox states, to achieve precise and controlled release kinetics [84]. For instance, pH-sensitive hydrogels are engineered to preferentially deliver their payload in acidic environments, making them particularly effective for targeting inflamed or infected tissues [45,75,85]. Similarly, thermoresponsive hydrogels undergo sol-gel phase transitions near physiological temperatures, allowing for minimally invasive administration as a liquid that solidifies upon reaching the target site [109,121].

Recent advances in thermoresponsive hydrogel architectures have introduced copolymer-based systems with enhanced tunability in drug release kinetics. In particular, copolymer brush coatings composed of PNIPAM and oligo(ethylene glycol) methacrylate (OEGMA) have demonstrated precise control over sol-gel transitions, making them highly attractive for LA applications [122,123]. By adjusting the hydrophilic-hydrophobic balance of these materials, researchers have successfully modulated LCST transitions, allowing for temperature-dependent drug adhesion and detachment mechanisms.

Furthermore, next-generation thermoresponsive hydrogels incorporating 2-hydroxyethyl methacrylate (HEMA) and PNIPAM or OEGMA have been developed to achieve rapid and reversible switching behavior [123]. These hybrid architectures enable fine-tuned control of anesthetic release, reducing burst release while extending drug retention at the target site. Such advancements suggest that integrating similar copolymer-based thermoresponsive hydrogels into LA delivery systems may further improve on-demand, site-specific drug release while minimizing systemic toxicity.

Dual- and multi-responsive systems further enhance the versatility of these hydrogels [16]. Combining pH and enzymatic sensitivity, for example, allows these systems to achieve superior precision in delivering LAs to specific tissue sites [94,107]. These advancements reduce systemic toxicity and enhance therapeutic efficacy, especially in complex surgical scenarios where localized anesthetic release is critical. Additionally, time-dependent release profiles in smart hydrogels enable pulsatile or sequential drug delivery tailored to patient-specific pain management needs [72,73,87].

Nanotechnology plays a pivotal role in advancing hydrogel-based LA systems [114]. Magnetic nanoparticles embedded within hydrogels enable external activation using magnetic fields to control drug release timing and dosage [92,124,125]. Similarly, light-responsive hydrogels utilize near-infrared light to trigger drug release, offering a non-invasive mechanism for modulating pain relief [72,91]. These innovations not only enhance precision but also open new avenues for integrating hydrogels with wearable or implantable technologies to enable continuous pain management.

As these advancements demonstrate, overcoming the obstacles associated with drug loading and sustained release in hydrogel-LA systems requires a multifaceted approach that combines cutting-edge materials science, computational tools, and responsive technologies. These advancements continue to redefine the possibilities in localized pain management, paving the way for more effective and patient-centered solutions.

### 6.2. Biocompatibility and Safety Concerns

Hydrogels have gained widespread recognition for their biocompatibility, a critical attribute for their application in localized drug delivery [14]. However, variations in polymer composition, crosslinking methods, and degradation products can pose significant safety concerns. For instance, synthetic hydrogels, while offering superior tunability, may release byproducts that provoke adverse tissue reactions or inflammation [126]. Synthetic hydrogels, in particular, may release degradation products that cause adverse tissue reactions [127]. In applications requiring chronic use or repeated administration, these risks necessitate careful evaluation and mitigation. Moreover, biocompatibility can differ markedly across tissue types, requiring tailored formulations for specific clinical scenarios [128].

To address these challenges, significant progress has been made in the use of natural polymers, such as hyaluronic acid and alginate, known for their inherent biocompatibility and low immunogenicity [129]. By combining these natural components with advanced synthetic materials, hybrid hydrogels are emerging as a promising approach to balance biocompatibility with functionality [15]. Innovations such as bioinert surface coatings and bioadhesive materials further enhance tissue compatibility while minimizing irritation, particularly in sensitive or inflamed tissues [130]. Long-term biocompatibility studies involving multi-organ assessments and detailed in vivo experiments will be essential in ensuring the safety of hydrogel-based systems.

Another promising strategy involves embedding immunomodulatory agents within hydrogels. By simultaneously delivering anti-inflammatory compounds alongside LAs, dual-functional hydrogels reduce immune responses while providing effective analgesia [131]. This approach has shown significant potential in a preclinical model, where the integration of immunomodulators enhanced tissue compatibility and therapeutic outcomes [132].

Biodegradability is another critical consideration for hydrogel-based systems. Biodegradable hydrogels engineered to break down into harmless residues remove the requirement for surgical extraction, enhancing patient convenience and safety [133]. Recent advancements in enzyme-sensitive and hydrolytically degradable materials allow for fine-tuned regulation of degradation rates, synchronizing drug release profiles with therapeutic needs. For instance, peptide-based hydrogels mimic the ECM, offering both structural integrity and controlled biodegradation, making them particularly effective for prolonged LA delivery [134].

Functionalized hydrogels have broadened the scope of their applications, integrating therapeutic agents beyond LAs to address complex clinical needs. These biofunctionalized systems co-deliver growth factors, anti-inflammatory agents, and regenerative molecules, facilitating not only pain relief but also accelerated tissue healing [135]. Multifunctional hydrogels have shown great promise in postoperative and regenerative medicine settings, where combined therapeutic effects significantly enhance recovery outcomes [136].

Adhesion to target tissues remains a critical challenge in achieving localized and sustained drug delivery. Innovations in bioadhesive hydrogels have provided solutions for dynamic and challenging surfaces, such as mucosal membranes or cartilage [137]. Enhanced adhesion minimizes drug leakage and off-target effects, improving therapeutic precision. Surface functionalization techniques, such as the incorporation of catechol groups, have demonstrated superior adhesion properties, even in moist and mechanically active environments [138].

Addressing biocompatibility and safety concerns requires a multidisciplinary approach that integrates materials science, pharmacology, and clinical insights. By prioritizing patient safety and therapeutic efficacy, hydrogel systems continue to evolve as transformative tools in LA delivery.

### 6.3. Mechanical Stability in Dynamic Environments

Hydrogels often encounter challenges with mechanical stability under physiological conditions, particularly in areas subjected to repetitive motion or mechanical stress. Insufficient structural integrity can result in premature degradation, limiting their effectiveness for delivering LAs in dynamic environments [139]. To address these limitations, researchers have developed advanced hydrogel systems that integrate nanotechnology and innovative crosslinking strategies, enabling hydrogels to maintain their structure while adapting to mechanical demands.

One prominent solution involves the development of nanocomposite hydrogels by incorporating reinforcing materials such as nanoparticles, nanosheets, or nanofibers. These additions significantly enhance the structural integrity of hydrogels, enabling them to withstand repetitive motion and stress in mechanically demanding regions such as joints or surgical sites [140]. For instance, graphene oxide-based hydrogels have demonstrated exceptional resilience, maintaining structural integrity and functionality over extended periods [141,142,143]. Specifically, polyacrylamide hydrogels crosslinked with graphene oxide-based crosslinkers exhibit a tensile strength of approximately 0.473 MPa and an elongation at break of around 1000%, indicating their robustness and flexibility [144]. Nanotechnology has also enabled the creation of hybrid systems where nanoparticles act as reservoirs within the hydrogel matrix [141]. These systems ensure sustained release while protecting the drug from degradation [142]. Additionally, the use of polymeric micelles and liposomes as nanocarriers embedded in hydrogels has shown promise in improving the solubility and stability of hydrophobic LAs [143,144]. These innovations are particularly valuable in complex anatomical regions, such as joints or surgical sites, where precise drug delivery is paramount.

Hybrid systems incorporating dynamic crosslinking mechanisms, such as reversible covalent bonds or supramolecular interactions, further enhance the adaptability of these hydrogels to mechanical challenges. These designs allow hydrogels to maintain their structure under stress while adapting to environmental changes [113]. For instance, hydrogels utilizing imine bonds have demonstrated significant mechanical resilience, with compressive strengths reaching up to 27.3 MPa and the ability to withstand compressive strains of approximately 98.4% before failure [145]. Such properties make them promising candidates for load-bearing applications where mechanical resilience is essential [146].

Shear-thinning hydrogels provide another innovative approach to improving mechanical stability. These materials exhibit reduced viscosity under mechanical stress, facilitating smooth injection and rapid recovery of structural integrity post-administration [147]. For instance, certain hydrogels demonstrate a decrease in viscosity from approximately 10 Pa·s to 0.1 Pa·s as the shear rate increases from 0.1 s^−1^ to 100 s^−1^, enabling their application in minimally invasive procedures [148]. Complementing these designs are self-healing hydrogels, which autonomously repair microdamage through reversible bonding mechanisms. This self-repair capability ensures consistent performance in dynamic environments, such as cartilage or wound sites, where repeated mechanical forces are unavoidable [149]. For example, certain self-healing hydrogels can restore up to 98.2% of their initial tensile stress and 92.6% of their initial strain after 24 h of healing, maintaining their mechanical integrity under cyclic loading conditions [150].

Externally activated hydrogels represent another significant advancement, allowing for precise control over both mechanical stability and therapeutic delivery. Magnetic nanoparticles embedded within hydrogels enable real-time modulation of drug release through external magnetic fields, offering clinicians the ability to adjust dosage and timing as needed [92,151]. Similarly, gold nanoparticle-based hydrogels have shown promise in photothermal therapy, where laser-induced localized heating enhances drug release while simultaneously providing therapeutic effects [72,91,143]. These externally triggered systems combine adaptability and precision, paving the way for patient-specific pain management strategies.

By leveraging these innovations, hydrogel-based systems are increasingly overcoming the mechanical challenges posed by physiological conditions. The integration of nanotechnology, responsive materials, and advanced engineering principles has transformed hydrogels into robust and adaptable platforms, ensuring their efficacy in delivering LAs in even the most demanding clinical scenarios.

### 6.4. 3D-Printed and Customized Hydrogel Systems

Advances in 3D printing have revolutionized hydrogel systems, allowing for the creation of highly personalized solutions tailored to individual patient needs [152]. These systems enable precise regulation of drug encapsulation, release profiles, and mechanical properties, making them adaptable to a wide range of clinical applications [153]. Additive manufacturing techniques allow researchers to fabricate hydrogels with complex geometries and spatially controlled drug distributions, opening new pathways for personalized medicine approaches in pain management.

The integration of computational modeling into 3D printing processes has further enhanced their capabilities. One key innovation in 3D-printed hydrogel systems is the integration of computational modeling into the design process. By simulating drug release patterns, mechanical stress responses, and degradation kinetics, researchers can optimize hydrogel performance before production [154]. This reduces the trial-and-error traditionally associated with hydrogel development, resulting in faster and more efficient clinical translation. For example, computationally optimized, site-specific hydrogels have demonstrated superior efficacy in preclinical models, providing sustained analgesia while minimizing systemic exposure [155]. These approaches not only enhance therapeutic outcomes but also support the development of hydrogel systems tailored to unique anatomical or pathological conditions.

The introduction of bioinks has further expanded the capabilities of 3D-printed hydrogels, allowing for the integration of living cells, growth factors, and other biological components directly into the hydrogel matrix. These biofunctionalized systems are particularly valuable in regenerative medicine, where pain relief and tissue repair must be addressed simultaneously [156]. For instance, hydrogels embedded with stem cells or regenerative growth factors have shown significant promise in accelerating wound healing and improving recovery outcomes [157]. Additionally, these bioinks enable the creation of hydrogels that replicate the ECM, creating an optimal environment for cell proliferation and differentiation while delivering sustained analgesia [158].

3D-printed hydrogels also offer unprecedented flexibility in designing systems for specific clinical scenarios. Multi-material printing techniques allow the fabrication of hydrogels with gradient properties, enabling controlled, sequential drug release or differential mechanical characteristics within a single construct [159]. Such designs are especially beneficial for treating complex pain conditions that require localized, time-dependent drug delivery [153].

Although progress has been made, obstacles still exist in scaling up the production of 3D-printed hydrogels for clinical applications. Ensuring consistent quality, reproducibility, and sterility in large-scale manufacturing is critical for regulatory approval and commercialization [160]. Moreover, the cost of advanced bioinks and specialized printing equipment can limit widespread adoption [161]. Addressing these barriers will require continued collaboration between material scientists, engineers, and clinicians to streamline production processes and reduce costs without compromising the performance of hydrogel systems.

### 6.5. Translational and Manufacturing Challenges

Despite promising preclinical advancements, the clinical translation of hydrogel-based LA systems encounters several obstacles. One of the most pressing challenges is scaling up production while maintaining product consistency, sterility, and quality control [162]. High costs associated with raw materials, complex synthesis processes, and specialized manufacturing facilities pose additional barriers [163]. Moreover, the intricate regulatory landscape for novel hydrogel technologies often prolongs the approval timeline, requiring extensive safety and efficacy data for clinical use [10].

To mitigate these hurdles, modular hydrogel systems with standardized components have gained attention for their adaptability across diverse clinical scenarios [164]. Automation technologies, such as continuous flow synthesis and 3D bioprinting, are driving advancements in cost-effective and scalable production [165]. These methods not only reduce variability in manufacturing but also allow for rapid prototyping and testing of hydrogel formulations tailored to specific applications [14].

Another promising strategy is the establishment of open-access libraries containing pre-validated hydrogel formulations [166]. These repositories can serve as valuable resources for researchers and regulatory agencies, expediting the approval process by leveraging existing safety data. Such libraries would also promote collaboration and reduce redundancies in hydrogel development efforts [167].

Partnerships among academic institutions, industries, and regulatory bodies will be essential to address these translational challenges. Establishing clear regulatory pathways that account for the distinctive characteristics of hydrogels, including their biodegradability and sophisticated drug-release mechanisms, will be critical [168]. Additionally, incorporating patient-centric metrics, such as quality-of-life assessments and long-term safety profiles, into clinical trials will provide a more comprehensive understanding of these systems’ real-world benefits [169].

### 6.6. Sustainability and Ethical Considerations

As the demand for hydrogel-based systems grows, their environmental and ethical implications must be carefully considered. The reliance on petrochemical-derived polymers in many hydrogel formulations raises sustainability and biodegradability concerns [169]. Developing eco-friendly alternatives, such as polymers derived from plant-based or biosynthetic sources, could significantly reduce the environmental impact of these systems [170]. These materials not only align with global sustainability goals but also enhance biodegradability, further addressing concerns about long-term accumulation in biological and environmental systems.

Ethical considerations, particularly regarding equitable access and inclusivity, are equally important. Hydrogel-based systems, while innovative, risk becoming inaccessible to underserved populations due to high production costs and limited availability [171]. Reducing these costs through advanced manufacturing techniques and government or industry subsidies could make these technologies more affordable and widely available. Clinical trials should also prioritize diversity in patient recruitment, ensuring that efficacy and safety data reflect a broad range of demographic and physiological factors [172].

Moreover, the ethical allocation of resources in developing and deploying hydrogel-based therapies must be transparent [173]. Addressing disparities in access to these advanced medical technologies will be essential for fostering trust and fairness as hydrogel systems become more prevalent in clinical settings

### 6.7. Interdisciplinary Collaboration and Future Directions

The successful integration of hydrogel-based LA systems into clinical practice requires interdisciplinary collaboration across materials science, pharmacology, and clinical medicine. Partnerships among academic researchers, industrial stakeholders, and regulatory agencies are crucial for addressing the complex challenges associated with these systems [174]. By pooling expertise and resources, such collaborations can streamline the development of standardized manufacturing protocols and evaluation criteria, ensuring the safety, efficacy, and scalability of hydrogel technologies.

Currently, hydrogel-based drug delivery systems primarily utilize clinically established LAs (e.g., bupivacaine, lidocaine, ropivacaine) rather than newly synthesized compounds. Most research efforts have focused on enhancing the solubility, stability, and controlled release of these existing anesthetics within hydrogel matrices [14,15,16]. However, the rational design of novel LA compounds specifically optimized for hydrogel formulations remains an open area for exploration. Future research may focus on modifying anesthetic structures to improve hydrogel compatibility, such as increasing hydrophilicity to reduce burst release or incorporating functional groups that interact with hydrogel polymer networks to enable sustained drug release. Computational modeling and high-throughput screening approaches may further facilitate the design of hydrogel-anesthetic pairings with superior pharmacokinetic and pharmacodynamic profiles [175].

Future developments in hydrogel-based LA delivery are likely to leverage multifunctional, stimuli-responsive copolymers that dynamically respond to physiological conditions. Schizophrenic copolymers, which exhibit reversible transitions between hydrophilic and hydrophobic states under temperature and pH changes, have recently been reported as promising candidates for biomedical applications [122]. These materials could enhance LA formulations by allowing temperature-triggered adhesion and detachment mechanisms, thereby optimizing local drug retention and release at surgical or pain-management sites.

Additionally, next-generation temperature-responsive systems could benefit from copolymer architectures similar to those developed in recent regenerative medicine studies, where P(NIPAM-co-HEMA) and P(OEGMA-co-HEMA) brushes demonstrated precise temperature-modulated adhesion and detachment [123]. By integrating such smart polymers into hydrogel-based LA systems, future formulations may achieve enhanced spatiotemporal control over anesthetic delivery, reducing systemic toxicity while improving therapeutic precision.

Digital health technologies represent an exciting frontier for hydrogel systems. Smart hydrogels embedded with biosensors or electronic interfaces offer the potential to monitor drug release, patient responses, and environmental conditions in real time [119]. Such feedback systems enable dynamic adjustment of drug release rates to align with changing clinical needs, significantly improving therapeutic outcomes [176]. For example, smart hydrogels integrated with wearable or implantable devices could allow clinicians to remotely monitor and adjust analgesic delivery, offering unprecedented precision in pain management [177,178].

Future advancements are expected to harness AI and machine learning for optimizing hydrogel design and function. These technologies could be used to predict drug release patterns, identify optimal polymer compositions, and even anticipate patient-specific therapeutic responses [116,179]. Such integration would enable highly personalized treatments, where hydrogel systems are tailored to individual pain management requirements.

Additionally, innovations in 3D printing and bioprinting are paving the way for customized hydrogel systems. By incorporating living cells, bioactive molecules, and multiple drug reservoirs, these systems can simultaneously address pain relief, tissue regeneration, and anti-inflammatory needs [153,180]. This multidisciplinary approach exemplifies the potential of hydrogel-based systems to revolutionize localized therapy while advancing the broader goals of personalized and regenerative medicine. Table 7 provides a consolidated summary of the challenges and corresponding innovations in hydrogel-based LA systems, underscoring the multifaceted advancements discussed in this section.

## 7. Conclusions

Hydrogel-based delivery systems for LAs represent a paradigm shift in pain management, addressing critical limitations of conventional formulations such as short duration of action, systemic toxicity, and frequent administration [15,16]. By encapsulating LAs within adaptable, biocompatible polymer networks, hydrogels provide prolonged, localized analgesia and reduce systemic exposure [10]. The ability of hydrogels to integrate stimuli-responsive release mechanisms further enhances their precision, aligning therapeutic effects with patient-specific needs while minimizing side effects [84,85].

Preclinical and clinical studies underscore the transformative potential of hydrogel-LA systems. Thermoresponsive hydrogels have been the most extensively studied in clinical settings, particularly in surgical applications, where they have demonstrated prolonged pain relief, reduced rescue analgesic use, and improved recovery outcomes. Stimuli-responsive hydrogels, such as enzyme- and pH-sensitive formulations, have shown promise in preclinical models by enabling site-specific release based on the local physiological environment. Multifunctional hydrogels, incorporating anti-inflammatory agents or regenerative properties, further extend the therapeutic scope of hydrogel-based LA delivery [71,92,107,151]. These advances align with modern medicine’s goals of holistic and patient-centric care, particularly in managing acute postoperative and chronic pain.

Despite these advancements, challenges remain. Issues such as burst release, mechanical instability in dynamic environments, and the scalability of production processes pose barriers to widespread clinical translation [69,181]. Addressing these challenges will require interdisciplinary collaboration, leveraging innovations in nanotechnology, 3D bioprinting, and AI to optimize design, production, and patient-specific applications [116,119,153,182,183]. Moreover, regulatory alignment and sustainability considerations are essential to ensure equitable access and long-term adoption of these technologies [168].

Looking ahead, combining hydrogels with wearable and implantable devices, coupled with advancements in precision medicine and real-time monitoring, is poised to redefine pain management [176,177]. These emerging solutions promise safer, more effective, and personalized therapies, transforming the clinical landscape of LAs. Continued innovation, supported by collaboration across scientific, clinical, and regulatory domains, will be instrumental in harnessing the complete capabilities of hydrogel-LA systems, pushing the boundaries of localized pain management and enhancing patient care.

## Figures and Tables

**Table 1:** Comparison of Natural and Synthetic Local Anesthetics.

	Cocaine	Procaine	Tetracaine	Lidocaine	Prilocaine	Bupivacaine	Ropivacaine
Year of Synthesis	1860 (Isolation), 1884 (First anesthetic use)	1905	1930	1943	1953	1957	1996
Chemical Structure	Natural alkaloid	Ester-based	Ester-based	Amide-based	Amide-based	Amide-based	Amide-based
Duration of Action	Short	Short	Long	Intermediate	Intermediate	Long	Long
Toxicity	High	Low	Moderate	Low	Low	Moderate (cardiotoxic)	Low
Therapeutic Use Cases	Topical anesthesia	Minor procedures	Spinal and ophthalmic anesthesia	Versatile, regional	Regional anesthesia, dental procedures	Epidurals, prolonged	Epidurals, safer alternative to bupivacaine
Key Limitations	Addictive, toxic	Short duration, slow onset	High systemic toxicity	Rare systemic toxicity	Risk of methemoglobinemia	High-dose cardiotoxicity	Requires higher doses for equivalent effect

**Table 2:** Evolution of Local Anesthetic Delivery Methods.

Delivery Method	Advantages	Limitations	Clinical Applications
Bolus Injection	Simple and quick to administer.	Short duration of action; requires frequent dosing, leading to potential cumulative toxicity.	Minor surgical procedures, dental anesthesia.
Continuous Infusion Pumps	Provides steady analgesia; reduces the need for frequent dosing.	Requires specialized equipment and meticulous monitoring; risk of infection and systemic toxicity.	Postoperative pain management, labor analgesia (epidurals).
Perineural Catheters	Delivers localized and sustained analgesia; reduces systemic drug exposure.	Risk of catheter dislodgement or infection; requires trained personnel for insertion and care.	Nerve blocks, postoperative pain control.
Liposomal Formulations	Controlled drug release over extended periods (up to 72 h); minimizes dosing frequency.	High production costs; inconsistent release profiles; limited stability in some formulations.	Outpatient surgeries, long-term pain relief in postoperative settings.
Hydrogel Systems	Controlled and sustained release; customizable for specific clinical needs; minimizes systemic toxicity.	High variability in formulation; challenges in scaling up for clinical use; requires further validation.	Emerging applications in postoperative and chronic pain management.

**Table 3:** Comparative Characteristics of Local Anesthetic Classes and Innovations.

	Ester-Based LAs	Amide-Based LAs	Emerging Trends
Examples	Procaine, Tetracaine	Lidocaine, Bupivacaine, Ropivacaine	Site-1 Sodium Channel Blockers, Peptide-Based Anesthetics, Injectable Hydrogels, Liposomal Bupivacaine
Metabolism	Plasma cholinesterases	Liver (cytochrome P450 enzymes)	Mechanism-specific (e.g., peptide degradation or stimuli-responsive release systems)
Duration of Action	Short	Moderate to long	Extended (e.g., liposomal formulations offer 72 h analgesia)
Adverse Effects	Higher risk of hypersensitivity (PABA byproduct)	Lower hypersensitivity risk; potential cardiotoxicity	Reduced systemic toxicity; improved specificity and safety
Clinical Utility	Limited to short-duration procedures	Versatile; suitable for a wide range of applications	Targeted, long-lasting analgesia; potential applications in personalized medicine
Mechanism of Action	Sodium channel blockade (conventional)	Sodium channel blockade (conventional)	Novel mechanisms (e.g., site-specific sodium channel inhibition or bioengineered peptide selectivity)
Advantages	Rapid metabolism reduces systemic accumulation	Lower hypersensitivity, stable pharmacokinetics	Prolonged action, site-specific delivery, reduced dosing frequency
Limitations	Short duration; hypersensitivity; rapid hydrolysis	Potential for toxicity at high doses	High production cost, complexity of design, variable stability (e.g., hydrogels, liposomes)

LA, local anesthetic; PABA, para-aminobenzoic acid.

**Table 4:** Key Mechanisms of Drug Release from Hydrogels.

Release Mechanism	Principle	Advantages	Limitations
Diffusion-controlled	Drug diffuses passively through hydrogel	Simple to design, effective for small molecules	Limited for large or hydrophobic drugs
Degradation-controlled	Matrix breakdown releases encapsulated drug	Tailorable release kinetics, site-specific	Requires precise control of degradation rate
Stimuli-responsive	Triggered by pH, temperature, or enzymes	Enables precision medicine, on-demand release	Requires external triggers or complex design

**Table 5:** Recent Preclinical Advancements in Hydrogel-Based Local Anesthetic Delivery.

Hydrogel Mechanism (As Reported in Studies)	Primary Polymer Used in Study	Local Anesthetic Used in Study	Key Findings	Reference
Thermoresponsive Hydrogels				
Thermosensitive, LCST-dependent phase transition	PNIPAM, LCST ~32 °C	Bupivacaine	Extended sensory blockade (~9 h) in sciatic nerve block model	[104]
Amphiphilic thermosensitive gelation	Pluronic F127 (PEO-PPO-PEO triblock copolymer)	Bupivacaine	Extended sensory (8.0 ± 1.6 h) and motor blockade (9.3 ± 1.6 h) in canine model	[71]
Amphiphilic thermoresponsive gelation	Poloxamer 407 (PEO-PPO-PEO triblock copolymer)	Lidocaine	Rapid onset; extended release; high mucoadhesion in wound healing models	[41]
PNIPAM copolymer-based gelation	PNDJ	Bupivacaine	Sustained analgesia (96 h), systemic bupivacaine release (>7 d) in rabbit knee surgery model	[74]
Stimuli-Responsive Hydrogels				
pH-sensitive swelling and drug release	Methylcellulose (Cellulose-derived polymer)	Bupivacaine	pH-sensitive release, prolonged analgesia (45% release over 48 h at pH 6.5 vs. 22% at pH 7.4)	[45]
Enzyme-triggered degradation	Polydopamine (Dopamine-derived polymer with enzyme-triggered degradation)	Lidocaine	Enzyme-responsive on-demand release, antibacterial activity, prolonged analgesic effects	[72]
Supramolecular interactions for sustained release	Alginate-based with phenolic nanofillers	Lidocaine	14-d sustained drug release via supramolecular interactions	[92]
Controlled network modulation	Bacterial cellulose (Cellulose-derived hydrogel with tunable network architecture)	Lidocaine	Controlled lidocaine release over 14 d through network architecture modulation	[105]
Multifunctional Hydrogels				
Thermosensitive wound-healing system	Carboxymethyl agarose-NIPAM copolymer	Lidocaine	Accelerated wound healing (97% improvement) and controlled drug release	[106]
Injectable dual-drug sequential release system	Biodegradable hydrogel with dexmedetomidine	Ropivacaine	Sequential drug release maintaining sensory (48 h) and motor blockade (36 h)	[73]
Gas-encapsulated controlled release	Pluronic F127-based system	Bupivacaine	Continuous release over 14 d, improved post-surgical mobility and collagen deposition	[86]
Immune-modulating hydrogel for tumor microenvironment	PLGA-based system with TLR7 agonist	Ropivacaine	Enhanced CD8+ T cell infiltration, reduced tumor recurrence, long-lasting analgesia	[70]
Dual-drug delivery for chemotherapy and analgesia	Pluronic F127 loaded with cisplatin and ropivacaine	Ropivacaine	Prolonged CIPNP pain relief (>10 h), increased CD8+ T cell infiltration, enhanced MHC-I expression	[75]
Hybrid & Self-Healing Hydrogels				
Self-healing hydrogel system with prolonged stability	Sodium deoxycholate-based system	Ropivacaine	Peripheral nerve block > 1 wk, structural recovery after mechanical disruption	[69]
pH-stabilizing hydrogel with controlled buffering	Calcium carbonate-bupivacaine system	Bupivacaine	Extended analgesic effects (44 h) by maintaining stable internal pH	[107]
Hybrid mucoadhesive system	Poloxamer-hyaluronic acid system	Bupivacaine/Ropivacaine	Optimized viscosity and micellar interactions for controlled release	[80]
Biodegradable nerve block hydrogel	Gelatin crosslinked with NHS-PEG-NHS	Bupivacaine	Porous structure for high drug loading, sustained release, reduced neurotoxicity	[40]
Nanoparticle-loaded topical anesthesia	Lidocaine-nanoparticle system	Lidocaine	Sustained lidocaine release, improved pain tolerance in vivo	[103]

PNIPAM, Poly(N-isopropylacrylamide); LCST, Lower critical solution temperature; PEO-PPO-PEO, Poly(ethylene oxide)-poly(propylene oxide)-poly(ethylene oxide); PNDJ, Poly(N-isopropylacrylamide-co-dimethylbutyrolactone acrylamide-co-Jeffamine M-1000 acrylamide); NIPAM, N-isopropylacrylamide; PLGA, Poly(lactic-co-glycolic acid); TLR7, Toll-like receptor 7; CIPNP, Chemotherapy-induced peripheral neuropathic pain; MHC, Major histocompatibility complex; NHS-PEG-NHS, N-hydroxysuccinimide-polyethylene glycol-N-hydroxysuccinimide.

**Table 6:** Summary of Key Clinical Studies on Hydrogel-Based Local Anesthetic Delivery Systems.

Study Focus	Hydrogel Type	Procedure Type	Sample Size (n)	Key Findings	Reference
Thermoresponsive Hydrogels					
Bimaxillary Surgery	PF72	Orthognathic Surgery	40	Significant reduction in NRS pain scores at 24 h (6.4 → 4.0) and 72 h (3.4 → 2.6); reduced rescue analgesic use (*p* < 0.05)	[96]
Laparoscopic Abdominal Surgery	PF72	Stomach/Colorectal Surgery	99	Lower cumulative NRS pain scores (135.3 vs. 188.7 AUC); no adverse events (*p* < 0.001); reduced systemic exposure	[109]
Minimally Invasive Colorectal Surgery	Poloxamer 407-Based Hydrogel	Laparoscopic Colorectal Surgery	61	Comparable analgesia to continuous infusion (*p* = 0.806); shorter hospital stay (7.2 ± 1.6 vs. 8.4 ± 2.8 d, *p* = 0.045)	[88]
Thoracoscopic Pulmonary Resection	Poloxamer 407-Based Hydrogel	Thoracic Surgery	89	Comparable fentanyl consumption (*p* = 0.37); easier administration; reduced systemic toxicity risks	[110]
Dental Socket Wound Delivery	Poloxamer-Polyelectrolyte Complex	Tooth Socket Wound Delivery	30	Rapid onset (46.5 ± 22.5 s); effective anesthesia for 202.5 ± 41.0 s; prolonged pain relief up to 48 h	[41]
Standard Hydrogels					
Dental Palatal Anesthesia	Liposomal Lidocaine-Prilocaine	Upper Molar Extractions	40	100% success rate vs. 40% failure for non-liposomal; longer anesthesia duration (26.8 ± 7.5 vs. 16.8 ± 4.8 min, *p* < 0.0001)	[111]
Topical Hydrogel Delivery Systems					
Topical Dermatological Application	Nonaqueous Drug-in-Matrix System	Topical Pain Management	15	Enhanced skin permeation; sustained analgesia over 12 h; no skin irritation	[112]

NRS, numerical rating scale; AUC, area under curve.

**Table 7:** Challenges and Innovations in Hydrogel-LA Systems.

Challenge	Description	Proposed Innovations
Drug Loading and Sustained Release	Difficulty in achieving optimal drug encapsulation without burst release or systemic toxicity.	Advanced crosslinking (e.g., reversible bonds), stimuli-responsive systems, nanocarriers, AI-based modeling, rational design of anesthetic compounds optimized for hydrogel compatibility, and next-generation thermoresponsive copolymer systems (e.g., PNIPAM-OEGMA, PNIPAM-HEMA) for precise LCST modulation and controlled anesthetic release.
Biocompatibility and Safety	Potential for cytotoxicity or inflammatory responses from synthetic materials and degradation products.	Use of natural polymers, bioinert surface coatings, immunomodulatory agents, and biodegradable hydrogels.
Mechanical Stability	Premature hydrogel degradation in dynamic environments (e.g., joints, surgical sites).	Nanocomposites (e.g., graphene oxide), self-healing hydrogels, and externally activated systems (e.g., magnetic nanoparticles).
Customization and 3D Printing	Challenges in creating patient-specific designs with consistent quality.	Computational modeling for optimization, bioinks for regenerative applications, and multi-material printing techniques.
Manufacturing and Scalability	High production costs, sterility issues, and lengthy regulatory approval processes.	Modular systems, continuous flow synthesis, open-access hydrogel libraries, and streamlined regulatory pathways.
Sustainability and Ethics	Environmental impact of petrochemical-derived polymers and equitable access for underserved populations.	Development of eco-friendly polymers, subsidies to reduce costs, and inclusion of diverse patient populations in clinical trials.
Interdisciplinary Collaboration	Need for integrated approaches across materials science, pharmacology, and medicine to accelerate innovation and implementation.	Partnerships between academia, industry, and regulators; integration of smart technologies (e.g., biosensors, AI).

PNIPAM, poly(N-isopropylacrylamide); OEGMA, oligo(ethylene glycol) methacrylate; HEMA, 2-hydroxyethyl methacrylate; LCST, lower critical solution temperature; AI, artificial intelligence.

## Data Availability

No new data were created or analyzed in this study. Data sharing is not applicable to this article.

## Abbreviations

The following abbreviations are used in this manuscript:

LA	Local anesthetic
LAST	Local anesthetic systemic toxicity
ECM	Extracellular matrix
PNIPAM	Poly(N-isopropylacrylamide)
LCST	Lower critical solution temperature
PNDJ	Poly(N-isopropylacrylamide-co-dimethylbutyrolactone acrylamide-co-Jeffamine M-1000 acrylamide)
PDA	Polydopamine
NRS	Numerical rating scale
AI	Artificial intelligence
OEGMA	Oligo(ethylene glycol) methacrylate
HEMA	2-hydroxyethyl methacrylate

## References

[1] Chen Q., Chen E., Qian X. (2021). A Narrative Review on Perioperative Pain Management Strategies in Enhanced Recovery Pathways—The Past, Present and Future. J. Clin. Med..

[2] Small C., Laycock H. (2020). Acute postoperative pain management. Br. J. Surg..

[3] Aboushaar N., Serrano N. (2024). The mutually reinforcing dynamics between pain and stress: Mechanisms, impacts and management strategies. Front. Pain Res..

[4] Vinyes D., Muñoz-Sellart M., Fischer L. (2023). Therapeutic Use of Low-Dose Local Anesthetics in Pain, Inflammation, and Other Clinical Conditions: A Systematic Scoping Review. J. Clin. Med..

[5] Getachew M., Tesfaye H., Yihunie W., Ayenew T., Alemu S., Dagnew E.M., Biyazin Y., Abebe D., Degefu N., Abebaw A. (2024). Sustained release local anesthetics for pain management: Relevance and formulation approaches. Front. Pain Res..

[6] Nestor C.C., Ng C., Sepulveda P., Irwin M.G. (2022). Pharmacological and clinical implications of local anaesthetic mixtures: A narrative review. Anaesthesia.

[7] Ilfeld B.M. (2017). Continuous Peripheral Nerve Blocks: An Update of the Published Evidence and Comparison With Novel, Alternative Analgesic Modalities. Anesth. Analg..

[8] Macfarlane A.J.R., Gitman M., Bornstein K.J., El-Boghdadly K., Weinberg G. (2021). Updates in our understanding of local anaesthetic systemic toxicity: A narrative review. Anaesthesia.

[9] On’Gele M.O., Weintraub S., Qi V., Kim J. (2024). Local Anesthetics, Local Anesthetic Systemic Toxicity (LAST), and Liposomal Bupivacaine. Anesth. Clin.

[10] Li Y., Owens G.E., Kohane D.S. (2023). Materials for Controlled Release of Local Anesthetics. ChemMedChem.

[11] Allen T.M., Cullis P.R. (2013). Liposomal drug delivery systems: From concept to clinical applications. Adv. Drug Deliv. Rev..

[12] Lambrechts M., O’Brien M.J., Savoie F.H., You Z. (2013). Liposomal extended-release bupivacaine for postsurgical analgesia. Patient Prefer. Adherence.

[13] Beach M.A., Nayanathara U., Gao Y., Zhang C., Xiong Y., Wang Y., Such G.K. (2024). Polymeric Nanoparticles for Drug Delivery. Chem. Rev..

[14] Hameed H., Faheem S., Paiva-Santos A.C., Sarwar H.S., Jamshaid M. (2024). A Comprehensive Review of Hydrogel-Based Drug Delivery Systems: Classification, Properties, Recent Trends, and Applications. AAPS PharmSciTech.

[15] Liu B., Chen K. (2024). Advances in Hydrogel-Based Drug Delivery Systems. Gels.

[16] Musuc A.M., Mititelu M., Chelu M. (2024). Hydrogel for Sustained Delivery of Therapeutic Agents. Gels.

[17] Zhang Y., Xu Y., Gao J. (2023). The engineering and application of extracellular matrix hydrogels: A review. Biomater. Sci..

[18] Zhou H., Zhu Y., Yang B., Huo Y., Yin Y., Jiang X., Ji W. (2024). Stimuli-responsive peptide hydrogels for biomedical applications. J. Mater. Chem. B.

[19] Visai L., Zorzetto L., Brambilla P., Bloise N., De Gregori M., Cobianchi L., Allegri M., Petrini P., Marcello E., Peloso A. (2016). From micro- to nanostructured implantable device for local anesthetic delivery. Int. J. Nanomed..

[20] Li M., He Y., Liu Z., Ma X., Sun F., Pei L., Ma C., Liu H., Ji T., Huang Y. (2023). Construction of meloxicam and bupivacaine co-delivery nanosystem based on the pathophysiological environment of surgical injuries for enhanced postoperative analgesia. Nano Res..

[21] Yu Y.-M., Long Y.-Z., Zhu Z.-Q. (2024). Chitosan, a Natural Polymer, is an Excellent Sustained-Release Carrier for Amide Local Anesthetics. J. Pain Res..

[22] Zhang W., Xu W., Ning C., Li M., Zhao G., Jiang W., Ding J., Chen X. (2018). Long-acting hydrogel/microsphere composite sequentially releases dexmedetomidine and bupivacaine for prolonged synergistic analgesia. Biomaterials.

[23] Narayanaswamy R., Torchilin V.P. (2019). Hydrogels and Their Applications in Targeted Drug Delivery. Molecules.

[24] Calatayud J., González A. (2003). History of the development and evolution of local anesthesia since the coca leaf. Anesthesiology.

[25] Koller C. (1892). The Sub-conjunctival application of Cocaine in Eye Operations. Trans. Am. Ophthalmol. Soc..

[26] Warner E.A. (1993). Cocaine abuse. Ann. Intern. Med..

[27] Whiteside J.B., Wildsmith J.A.W. (2001). Developments in local anaesthetic drugs. Br. J. Anaesth..

[28] Tobe M., Suto T., Saito S. (2018). The history and progress of local anesthesia: Multiple approaches to elongate the action. J. Anesth..

[29] Zimmer M. (2014). History of anaesthesia: Early forms of local anaesthesia. Eur. J. Anaesthesiol..

[30] Drasner K., Eger I., Saidman L.J., Westhorpe R.N. (2014). The Development of Local Anesthetics. The Wondrous Story of Anesthesia.

[31] Butterworth J.F.t., Strichartz G.R. (1990). Molecular mechanisms of local anesthesia: A review. Anesthesiology.

[32] Ruetsch Y.A., Böni T., Borgeat A. (2001). From cocaine to ropivacaine: The history of local anesthetic drugs. Curr. Top Med. Chem..

[33] Albrecht E., Chin K.J. (2020). Advances in regional anaesthesia and acute pain management: A narrative review. Anaesthesia.

[34] Moore J.M. (2009). Continuous spinal anesthesia. Am. J. Ther..

[35] Toledano R.D., Tsen L.C. (2014). Epidural catheter design: History, innovations, and clinical implications. Anesthesiology.

[36] Jeng C.L., Torrillo T.M., Rosenblatt M.A. (2010). Complications of peripheral nerve blocks. Br. J. Anaesth..

[37] Ilfeld B.M., Eisenach J.C., Gabriel R.A. (2021). Clinical Effectiveness of Liposomal Bupivacaine Administered by Infiltration or Peripheral Nerve Block to Treat Postoperative Pain. Anesthesiology.

[38] Hamilton T.W., Athanassoglou V., Mellon S., Strickland L.H., Trivella M., Murray D., Pandit H.G. (2017). Liposomal bupivacaine infiltration at the surgical site for the management of postoperative pain. Cochrane. Database. Syst. Rev..

[39] Aggarwal N. (2018). Local anesthetics systemic toxicity association with exparel (bupivacaine liposome)- a pharmacovigilance evaluation. Expert. Opin. Drug Saf..

[40] Zhang Q., Liu X., Liu H., Li S., An Z., Feng Z. (2024). Construction of bupivacaine-loaded gelatin-based hydrogel delivery system for sciatic nerve block in mice. J. Biomed. Mater. Res. A.

[41] Supachawaroj N., Limsitthichaikoon S. (2024). Lidocaine HCl-Loaded Polyelectrolyte Complex -Poloxamer Thermoresponsive Hydrogel: In Vitro- In Vivo Anesthetic Evaluations for Tooth Socket Wound Delivery. AAPS PharmSciTech.

[42] Tang C., Miller A.F., Saiani A. (2014). Peptide hydrogels as mucoadhesives for local drug delivery. Int. J. Pharm..

[43] Zhou C., Huang J., Yang Q., Li T., Liu J., Qian Z. (2018). Gold nanorods-based thermosensitive hydrogel produces selective long-lasting regional anesthesia triggered by photothermal activation of Transient Receptor Potential Vanilloid Type-1 channels. Colloids Surf. B Biointerfaces.

[44] Kurian A.G., Singh R.K., Sagar V., Lee J.-H., Kim H.-W. (2024). Nanozyme-Engineered Hydrogels for Anti-Inflammation and Skin Regeneration. Nano Micro. Lett..

[45] Wójcik-Pastuszka D., Frąk A., Musiał W. (2024). Influence of the Acceptor Fluid on the Bupivacaine Release from the Prospective Intra-Articular Methylcellulose Hydrogel. Pharmaceutics.

[46] Ma X., Sekhar K.P.C., Zhang P., Cui J. (2024). Advances in stimuli-responsive injectable hydrogels for biomedical applications. Biomater. Sci..

[47] Martins C.F., García-Astrain C., Conde J., Liz-Marzán L.M. (2024). Nanocomposite hydrogel microneedles: A theranostic toolbox for personalized medicine. Drug Deliv. Transl. Res..

[48] Taylor A., McLeod G. (2020). Basic pharmacology of local anaesthetics. BJA Educ..

[49] Golembiewski J. (2013). Local anesthetics. J. Perianesth. Nurs..

[50] Ring M.E. (2007). The History of Local Anesthesia. J. Calif. Dent. Assoc..

[51] Moore P.A., Hersh E.V. (2010). Local anesthetics: Pharmacology and toxicity. Dent. Clin. N. Am..

[52] Bourne E., Wright C., Royse C. (2010). A review of local anesthetic cardiotoxicity and treatment with lipid emulsion. Local Reg. Anesth..

[53] Casati A., Santorsola R., Cerchierini E., Moizo E. (2001). Ropivacaine. Minerva. Anestesiol..

[54] Becker D.E., Reed K.L. (2012). Local Anesthetics: Review of Pharmacological Considerations. Anesth. Prog..

[55] Kaufman E., Goharian S., Katz Y. (2000). Adverse reactions triggered by dental local anesthetics: A clinical survey. Anesth. Prog..

[56] Deleon A.M., Wong C.A. (2010). Levobupivacaine versus bupivacaine: Is there as winner?. Minerva. Anestesiol..

[57] Desai N., Kirkham K.R., Albrecht E. (2021). Local anaesthetic adjuncts for peripheral regional anaesthesia: A narrative review. Anaesthesia.

[58] Lirk P., Hollmann M.W., Strichartz G. (2018). The Science of Local Anesthesia: Basic Research, Clinical Application, and Future Directions. Anesth. Analg..

[59] Maqusood S., Madavi S., Bele A., Dash S., Bawiskar D. (2024). Pharmacological Insights of Ropivacaine and Clinical Applications: A Narrative Review. Cureus.

[60] Hoegberg L.C., Bania T.C., Lavergne V., Bailey B., Turgeon A.F., Thomas S.H., Morris M., Miller-Nesbitt A., Mégarbane B., Magder S. (2016). Systematic review of the effect of intravenous lipid emulsion therapy for local anesthetic toxicity. Clin. Toxicol..

[61] Rosenberg P.H., Veering B.T., Urmey W.F. (2004). Maximum recommended doses of local anesthetics: A multifactorial concept. Reg. Anesth. Pain Med..

[62] Song S., Xia X., Shorty T., Li T., Stevens A.O., Zhao C., He Y. (2024). Molecular Dynamics Insights into Peptide-Based Tetrodotoxin Delivery Nanostructures. Molecules.

[63] Liu J., Peng F., Kang Y., Gong D., Fan J., Zhang W., Qiu F. (2021). High-Loading Self-Assembling Peptide Nanoparticles as a Lipid-Free Carrier for Hydrophobic General Anesthetics. Int. J. Nanomed..

[64] King C.H., Beutler S.S., Kaye A.D., Urman R.D. (2017). Pharmacologic Properties of Novel Local Anesthetic Agents in Anesthesia Practice. Anesth. Clin..

[65] Wang D., Li Y., Deng X., Torre M., Zhang Z., Li X., Zhang W., Cullion K., Kohane D.S., Weldon C.B. (2023). An aptamer-based depot system for sustained release of small molecule therapeutics. Nat. Commun..

[66] Priya A.S., Premanand R., Ragupathi I., Bhaviripudi V.R., Aepuru R., Kannan K., Shanmugaraj K. (2024). Comprehensive Review of Hydrogel Synthesis, Characterization, and Emerging Applications. J. Compos. Sci..

[67] Cao H., Duan L., Zhang Y., Cao J., Zhang K. (2021). Current hydrogel advances in physicochemical and biological response-driven biomedical application diversity. Signal Transduct. Target. Ther..

[68] Kaith B.S., Singh A., Sharma A.K., Sud D. (2021). Hydrogels: Synthesis, Classification, Properties and Potential Applications—A Brief Review. J. Polym. Environ..

[69] Tan X., Zhou Y., Qin Y., Wu L., Yang R., Bao X., Jiang R., Sun X., Ying X., Ben Z. (2024). Self-Healing Hydrogel Resulting from the Noncovalent Interaction between Ropivacaine and Low-Molecular-Weight Gelator Sodium Deoxycholate Achieves Stable and Endurable Local Analgesia in Vivo. ACS Appl. Mater. Interfaces.

[70] Zhao M., Zhu S., Zhang D., Zhou C., Yang Z., Wang C., Liu X., Zhang J. (2023). Long-lasting postoperative analgesia with local anesthetic-loaded hydrogels prevent tumor recurrence via enhancing CD8(+)T cell infiltration. J. Nanobiotechnol..

[71] Kim J., Kim D., Shin D., Sung T., Rhee S., Kim M., Nam C., Lee I., Son W.G. (2023). Effect of temperature-responsive hydrogel on femoral and sciatic nerve blocks using bupivacaine in Beagle dogs. Vet. Med. Sci..

[72] Wu Y., Lin Y., Chen Y., Fan H., Zhang J., Li J., Lin W., Yi G., Feng X. (2024). Adhesive polydopamine-based photothermal hybrid hydrogel for on-demand lidocaine delivery, effective anti-bacteria, and prolonged local long-lasting analgesia. Int. J. Biol. Macromol..

[73] Li Y., Chen Y., Xue Y., Jin J., Xu Y., Zeng W., Liu J., Xie J. (2024). Injectable Hydrogel Delivery System with High Drug Loading for Prolonging Local Anesthesia. Adv. Sci..

[74] Overstreet D.J., Zdrale G., McLaren A.C. (2024). Extended Release of Bupivacaine from Temperature-Responsive PNDJ Hydrogels Improves Postoperative Weight-Bearing in Rabbits Following Knee Surgery. Pharmaceuticals.

[75] Qing X., Dou R., Wang P., Zhou M., Cao C., Zhang H., Qiu G., Yang Z., Zhang J., Liu H. (2023). Ropivacaine-loaded hydrogels for prolonged relief of chemotherapy-induced peripheral neuropathic pain and potentiated chemotherapy. J. Nanobiotechnol..

[76] Bustamante-Torres M., Romero-Fierro D., Arcentales-Vera B., Palomino K., Magaña H., Bucio E. (2021). Hydrogels Classification According to the Physical or Chemical Interactions and as Stimuli-Sensitive Materials. Gels.

[77] Zhao L., Zhou Y., Zhang J., Liang H., Chen X., Tan H. (2023). Natural Polymer-Based Hydrogels: From Polymer to Biomedical Applications. Pharmaceutics.

[78] Van Sprang J.F., Smits I.P.M., Nooten J.C.H., Fransen P.-P.K.H., Söntjens S.H.M., Van Houtem M.H.C.J., Janssen H.M., Rutten M.G.T.A., Schotman M.J.G., Dankers P.Y.W. (2025). From natural to synthetic hydrogels: How much biochemical complexity is required for mechanotransduction?. J. Mater. Chem. B.

[79] Zhong Y., Lin Q., Yu H., Shao L., Cui X., Pang Q., Zhu Y., Hou R. (2024). Construction methods and biomedical applications of PVA-based hydrogels. Front. Chem..

[80] Sepulveda A.F., Kumpgdee-Vollrath M., Franco M., Yokaichiya F., de Araujo D.R. (2023). Supramolecular structure organization and rheological properties modulate the performance of hyaluronic acid-loaded thermosensitive hydrogels as drug-delivery systems. J. Colloid Interface Sci..

[81] Liu Y., Islam M.S., Bakker A., Li Z., Ajam A., Kruzic J.J., Kilian K.A. (2025). Improving the bioactivity and mechanical properties of poly(ethylene glycol)-based hydrogels through a supramolecular support network. J. Mater. Chem. B.

[82] Liu J., Du C., Huang W., Lei Y. (2024). Injectable smart stimuli-responsive hydrogels: Pioneering advancements in biomedical applications. Biomater. Sci..

[83] Wang J., Yang W., Li Y., Ma X., Xie Y., Zhou G., Liu S. (2024). Dual-Temperature/pH-Sensitive Hydrogels with Excellent Strength and Toughness Crosslinked Using Three Crosslinking Methods. Gels.

[84] Zhang Y., Wu B.M. (2023). Current Advances in Stimuli-Responsive Hydrogels as Smart Drug Delivery Carriers. Gels.

[85] Morozova S.M., Korzhikova-Vlakh E.G. (2023). Fibrillar Hydrogel Based on Cellulose Nanocrystals Crosslinked via Diels-Alder Reaction: Preparation and pH-Sensitive Release of Benzocaine. Pharmaceuticals.

[86] Yu Y.H., Lee C.H., Hsu Y.H., Chou Y.C., Hong B.K., Huang C.T., Liu S.J. (2023). Novel CO(2)-encapsulated Pluronic F127 hydrogel for the treatment of Achilles tendon injury. Sci. Rep..

[87] Choi W., Aizik G., Ostertag-Hill C.A., Kohane D.S. (2024). A hybrid nanoparticle-protein hydrogel system for prolonged local anesthesia. Biomaterials.

[88] Shin J.K., Jeong H., Lee W.Y., Yun S.H., Cho Y.B., Huh J.W., Park Y.A., Sim W.S., Kim H.C. (2022). Efficacy of a local anesthetic gel infusion kit for pain relief after minimally invasive colorectal surgery: An open-label, randomized clinical trial. Sci. Rep..

[89] Croitoru C., Roata I.C., Pascu A., Stanciu E.M. (2020). Diffusion and Controlled Release in Physically Crosslinked Poly (Vinyl Alcohol)/Iota-Carrageenan Hydrogel Blends. Polymers.

[90] Tran Vo T.M., Nakajima K., Potiyaraj P., Kobayashi T. (2024). In situ sono-rheometric assessment of procaine-loaded calcium pectinate hydrogel for enhanced drug releasing under ultrasound stimulation. Int. J. Biol. Macromol..

[91] Zhang J., Zhu S., Zhao M., Zhou M., Zhu X., Qing X., Yang Z., Wei P., Zhang G., He W. (2023). Analgesic and potentiated photothermal therapy with ropivacaine-loaded hydrogels. Theranostics.

[92] Pan J., Liao H., Gong G., He Y., Wang Q., Qin L., Zhang Y., Ejima H., Tardy B.L., Richardson J.J. (2023). Supramolecular nanoarchitectonics of phenolic-based nanofiller for controlled diffusion of versatile drugs in hydrogels. J. Control. Release.

[93] Thang N.H., Chien T.B., Cuong D.X. (2023). Polymer-Based Hydrogels Applied in Drug Delivery: An Overview. Gels.

[94] Deng W., Chen J., Wang X., Wang Q., Zhao L., Zhu Y., Yan J., Zheng Y. (2024). Paravertebrally-Injected Multifunctional Hydrogel for Sustained Anti-Inflammation and Pain Relief in Lumbar Disc Herniation. Adv. Health Mater..

[95] Tanga S., Aucamp M., Ramburrun P. (2023). Injectable Thermoresponsive Hydrogels for Cancer Therapy: Challenges and Prospects. Gels.

[96] Yun C.W., Kim K.H., Lee W., Kim S.H. (2024). Comparative Analysis of Temperature-Responsive Hydrogel (PF 72) for Postoperative Pain After Bimaxillary Surgery: A Retro-spective Study. Aesthetic Plast. Surg..

[97] Manghnani P.N., Nelson A.Z., Wong K., Lee Y.W., Khan S.A., Doyle P.S. (2025). From burst to controlled release: Using hydrogel crosslinking chemistry to tune release of micro-crystalline active pharmaceutical ingredients. RSC Pharm..

[98] Rizwan M., Yahya R., Hassan A., Yar M., Azzahari A., Selvanathan V., Sonsudin F., Abouloula C. (2017). pH Sensitive Hydrogels in Drug Delivery: Brief History, Properties, Swelling, and Release Mechanism, Material Selection and Applications. Polymers.

[99] Lavrentev F.V., Shilovskikh V.V., Alabusheva V.S., Yurova V.Y., Nikitina A.A., Ulasevich S.A., Skorb E.V. (2023). Diffusion-Limited Processes in Hydrogels with Chosen Applications from Drug Delivery to Electronic Components. Molecules.

[100] Amiri N., Ghaffari S., Hassanpour I., Chae T., Jalili R., Kilani R.T., Ko F., Ghahary A., Lange D. (2023). Antibacterial Thermosensitive Silver–Hydrogel Nanocomposite Improves Wound Healing. Gels.

[101] Ma H., Pan Z., Lai B., Zan C., Liu H. (2023). Recent Research Advances in Nano-Based Drug Delivery Systems for Local Anesthetics. Drug Des. Dev. Ther..

[102] Fomina Y.S., Semkina A.S., Zagoskin Y.D., Aleksanyan M.M., Chvalun S.N., Grigoriev T.E. (2023). Biocompatible Hydrogels Based on Biodegradable Polyesters and Their Copolymers. Colloid J..

[103] Gergen A.K., Madsen H.J., Rocker A.J., White A.M., Jones K., Merrick D.T., Park D., Rove J.Y. (2024). Making a Painless Drain: Proof of Concept. Semin. Thorac. Cardiovasc. Surg..

[104] Hoare T., Young S., Lawlor M.W., Kohane D.S. (2012). Thermoresponsive nanogels for prolonged duration local anesthesia. Acta Biomater..

[105] Amorim J., Liao K., Mandal A., Costa A.F.S., Roumeli E., Sarubbo L.A. (2024). Impact of Carbon Source on Bacterial Cellulose Network Architecture and Prolonged Lidocaine Release. Polymers.

[106] Cai Y., Xin L., Sun P., Li H., Liu C., Fang L. (2023). Temperature-sensitive multifunctional intelligent responsive hydrogel based on carboxymethyl agarose and N-isopropylacrylamide: Controlled drug release and accelerated wound healing. Carbohydr. Polym..

[107] Guo W., Cao D., Rao W., Sun T., Wei Y., Wang Y., Yu L., Ding J. (2023). Achieving Long-Acting Local Analgesia Using an Intelligent Hydrogel Encapsulated with Drug and pH Regulator. ACS Appl. Mater. Interfaces.

[108] Patel P., Mandal A., Gote V., Pal D., Mitra A.K. (2019). Thermosensitive hydrogel-based drug delivery system for sustained drug release. J. Polym. Res..

[109] Choi B.M., Hwang C.S., Yoon Y.S., Park I.J., Yoo M.W., Kim B.S. (2022). Novel temperature-responsive hydrogel injected to the incision site for postoperative pain relief in laparoscopic abdominal surgery: A single-blind, randomized, pivotal clinical trial. Surg. Endosc..

[110] Jeon J.H., Seong Y.W., Han J.E., Cho S., Kim J.H., Jheon S., Kim K. (2022). Randomized Trial of Poloxamer 407-Based Ropivacaine Hydrogel After Thoracoscopic Pulmonary Resection. Ann. Thorac. Surg..

[111] Amorim K.S., Franz-Montan M., Groppo F.C., Muniz B.V., Araújo J.S.M., Santana J.V.F., Dantas A., de Paula E., Souza L.M.A. (2020). Palatal needle-free anesthesia for upper molars extraction. A randomized clinical trial. J. Craniomaxillofac. Surg..

[112] Greuber E., Vought K., Patel K., Suzuki H., Usuda K., Shiramizu A., Koplowitz L.P., Koplowitz B., Maibach H.I., Lissin D. (2021). Biorelevant In Vitro Skin Permeation Testing and In Vivo Pharmacokinetic Characterization of Lidocaine from a Nonaqueous Drug-in-Matrix Topical System. AAPS PharmSciTech.

[113] Hu W., Wang Z., Xiao Y., Zhang S., Wang J. (2019). Advances in crosslinking strategies of biomedical hydrogels. Biomater. Sci..

[114] Valentino A., Yazdanpanah S., Conte R., Calarco A., Peluso G. (2024). Smart Nanocomposite Hydrogels as Next-Generation Therapeutic and Diagnostic Solutions. Gels.

[115] Hussain AL-Mayahy M., Imad Hameed H. (2023). Hydrogels and Nanogels as a Promising Carrier for Drug Delivery. Hydrogels and Nanogels—Applications in Medicine.

[116] Negut I., Bita B. (2023). Exploring the Potential of Artificial Intelligence for Hydrogel Development—A Short Review. Gels.

[117] Sheth S., Barnard E., Hyatt B., Rathinam M., Zustiak S.P. (2019). Predicting Drug Release From Degradable Hydrogels Using Fluorescence Correlation Spectroscopy and Mathematical Modeling. Front. Bioeng. Biotechnol..

[118] Bediaga-Bañeres H., Moreno-Benítez I., Arrasate S., Pérez-Álvarez L., Halder A.K., Cordeiro M.N.D.S., González-Díaz H., Vilas-Vilela J.L. (2025). Artificial Intelligence-Driven Modeling for Hydrogel Three-Dimensional Printing: Computational and Experimental Cases of Study. Polymers.

[119] Tian B., Liu J. (2023). Smart stimuli-responsive chitosan hydrogel for drug delivery: A review. Int J Biol Macromol.

[120] Zinkovska N., Smilek J., Pekar M. (2020). Gradient Hydrogels—The State of the Art in Preparation Methods. Polymers.

[121] Heffernan J.M., McLaren A.C., Glass C.M., Overstreet D.J. (2023). Extended Release of Bupivacaine from Temperature-responsive Hydrogels Provides Multi-day Analgesia for Postoperative Pain. Pain Med..

[122] Shymborska Y., Stetsyshyn Y., Awsiuk K., Raczkowska J., Bernasik A., Janiszewska N., Da Bczyński P., Kostruba A., Budkowski A. (2023). Temperature- and pH-Responsive Schizophrenic Copolymer Brush Coatings with Enhanced Temperature Response in Pure Water. ACS Appl. Mater. Interfaces.

[123] Tymetska S., Shymborska Y., Stetsyshyn Y., Budkowski A., Bernasik A., Awsiuk K., Donchak V., Raczkowska J. (2023). Thermoresponsive Smart Copolymer Coatings Based on P(NIPAM-co-HEMA) and P(OEGMA-co-HEMA) Brushes for Regenerative Medicine. ACS Biomater. Sci. Eng..

[124] Peng F., Liu J., Zhang Y., Fan J., Gong D., He L., Zhang W., Qiu F. (2022). Designer self-assembling peptide nanofibers induce biomineralization of lidocaine for slow-release and prolonged analgesia. Acta Biomater..

[125] Deng W., Yan Y., Zhuang P., Liu X., Tian K., Huang W., Li C. (2022). Synthesis of nanocapsules blended polymeric hydrogel loaded with bupivacaine drug delivery system for local anesthetics and pain management. Drug Deliv..

[126] Zafar S., Hanif M., Azeem M., Mahmood K., Gondal S.A. (2022). Role of crosslinkers for synthesizing biocompatible, biodegradable and mechanically strong hydrogels with desired release profile. Polym. Bull..

[127] Ramot Y., Haim-Zada M., Domb A.J., Nyska A. (2016). Biocompatibility and safety of PLA and its copolymers. Adv. Drug Deliv. Rev..

[128] Fan Y., Wang H., Wang C., Xing Y., Liu S., Feng L., Zhang X., Chen J. (2024). Advances in Smart-Response Hydrogels for Skin Wound Repair. Polymers.

[129] Satchanska G., Davidova S., Petrov P.D. (2024). Natural and Synthetic Polymers for Biomedical and Environmental Applications. Polymers.

[130] Mir M., Ali M.N., Barakullah A., Gulzar A., Arshad M., Fatima S., Asad M. (2018). Synthetic polymeric biomaterials for wound healing: A review. Prog. Biomater..

[131] Liu X., Hu Y., Ju Y., Yang P., Shen N., Yang A., Wu R., Fang B., Liu L. (2024). Immunomodulatory hydrogels for tissue repair and regeneration. APL Mater..

[132] Grindy S., Gil D., Suhardi J., Fan Y., Moore K., Hugard S., Leape C., Randolph M., Asik M.D., Muratoglu O. (2023). Hydrogel device for analgesic drugs with in-situ loading and polymerization. J. Control. Release.

[133] Garcia-Garcia A., Muñana-González S., Lanceros-Mendez S., Ruiz-Rubio L., Alvarez L.P., Vilas-Vilela J.L. (2024). Biodegradable Natural Hydrogels for Tissue Engineering, Controlled Release, and Soil Remediation. Polymers.

[134] Xu L., Qiu L., Sheng Y., Sun Y., Deng L., Li X., Bradley M., Zhang R. (2018). Biodegradable pH-responsive hydrogels for controlled dual-drug release. J. Mater. Chem. B.

[135] Chelu M., Magdalena Musuc A. (2024). Biomaterials-Based Hydrogels for Therapeutic Applications. Biomaterials in Microencapsulation.

[136] Sobczak M. (2022). Enzyme-Responsive Hydrogels as Potential Drug Delivery Systems—State of Knowledge and Future Prospects. Int. J. Mol. Sci..

[137] Xiong Y., Zhang X., Ma X., Wang W., Yan F., Zhao X., Chu X., Xu W., Sun C. (2021). A review of the properties and applications of bioadhesive hydrogels. Polym. Chem..

[138] Karami P., Martin R., Laurent A., Nam H.Y., Philippe V., Applegate L.A., Pioletti D.P. (2024). An Adhesive Hydrogel Technology for Enhanced Cartilage Repair: A Preliminary Proof of Concept. Gels.

[139] Rumon M.M.H., Rahman M.S., Akib A.A., Sohag M.S., Rakib M.R.A., Khan M.A.R., Yesmin F., Shakil M.S., Rahman Khan M.M. (2025). Progress in hydrogel toughening: Addressing structural and crosslinking challenges for biomedical applications. Discov. Mater..

[140] Baishya G., Parasar B., Limboo M., Kumar R., Dutta A., Hussain A., Phukan M.M., Saikia D. (2024). Advancements in nanocomposite hydrogels: A comprehensive review of biomedical applications. Discov. Mater..

[141] Luu C.H., Nguyen G., Le T.T., Nguyen T.N., Giang Phan V.H., Murugesan M., Mathiyalagan R., Jing L., Janarthanan G., Yang D.C. (2022). Graphene Oxide-Reinforced Alginate Hydrogel for Controlled Release of Local Anesthetics: Synthesis, Characterization, and Release Studies. Gels.

[142] Al Homsi R., Eltahir S., Jagal J., Ali Abdelkareem M., Ghoneim M.M., Rawas-Qalaji M.M., Greish K., Haider M. (2022). Thermosensitive injectable graphene oxide/chitosan-based nanocomposite hydrogels for controlling the in vivo release of bupivacaine hydrochloride. Int. J. Pharm..

[143] Li W., Zhang G., Wei X. (2021). Lidocaine-loaded reduced graphene oxide hydrogel for prolongation of effects of local anesthesia: In vitro and in vivo analyses. J. Biomater. Appl..

[144] Rumon M.M.H., Sarkar S.D., Uddin M.M., Alam M.M., Karobi S.N., Ayfar A., Azam M.S., Roy C.K. (2022). Graphene oxide based crosslinker for simultaneous enhancement of mechanical toughness and self-healing capability of conventional hydrogels. RSC Adv..

[145] Liu Y., Liu J., Yang H., Liu K., Miao R., Peng H., Fang Y. (2018). Dynamic covalent bond-based hydrogels with superior compressive strength, exceptional slice-resistance and self-healing properties. Soft Matter.

[146] Sahajpal K., Shekhar S., Kumar A., Sharma B., Meena M.K., Bhagi A.K., Sharma S. (2022). Dynamic protein and polypeptide hydrogels based on Schiff base co-assembly for biomedicine. J. Mater. Chem. B.

[147] Zandi N., Sani E.S., Mostafavi E., Ibrahim D.M., Saleh B., Shokrgozar M.A., Tamjid E., Weiss P.S., Simchi A., Annabi N. (2021). Nanoengineered shear-thinning and bioprintable hydrogel as a versatile platform for biomedical applications. Biomaterials.

[148] Chen M.H., Wang L.L., Chung J.J., Kim Y.H., Atluri P., Burdick J.A. (2017). Methods To Assess Shear-Thinning Hydrogels for Application As Injectable Biomaterials. ACS Biomater. Sci. Eng..

[149] Cui H., Cui B., Chen H., Geng X., Geng X., Li Z., Cao S., Shen J., Li J. (2023). A chitosan-based self-healing hydrogel for accelerating infected wound healing. Biomater. Sci..

[150] Li Z., Lu J., Ji T., Xue Y., Zhao L., Zhao K., Jia B., Wang B., Wang J., Zhang S. (2024). Self-Healing Hydrogel Bioelectronics. Adv. Mater..

[151] Borges R., Kai K.C., Lima C.A., Zezell D.M., de Araujo D.R., Marchi J. (2021). Bioactive glass/poloxamer 407 hydrogel composite as a drug delivery system: The interplay between glass dissolution and drug release kinetics. Colloids Surf. B Biointerfaces.

[152] Liang X., Zhang M., Chong C.-M., Lin D., Chen S., Zhen Y., Ding H., Zhong H.-J. (2024). Recent Advances in the 3D Printing of Conductive Hydrogels for Sensor Applications: A Review. Polymers.

[153] Omidian H., Mfoafo K. (2024). Three-Dimensional Printing Strategies for Enhanced Hydrogel Applications. Gels.

[154] Agrawal A., Hussain C.M. (2023). 3D-Printed Hydrogel for Diverse Applications: A Review. Gels.

[155] Uchida D.T., Bruschi M.L. (2023). 3D Printing as a Technological Strategy for the Personalized Treatment of Wound Healing. AAPS PharmSciTech.

[156] Gopinathan J., Noh I. (2018). Recent trends in bioinks for 3D printing. Biomater. Res..

[157] Sousa A.C., McDermott G., Shields F., Alvites R., Lopes B., Sousa P., Moreira A., Coelho A., Santos J.D., Atayde L. (2024). Innovative Ink-Based 3D Hydrogel Bioprinted Formulations for Tissue Engineering Applications. Gels.

[158] Wang H., Yu H., Zhou X., Zhang J., Zhou H., Hao H., Ding L., Li H., Gu Y., Ma J. (2022). An Overview of Extracellular Matrix-Based Bioinks for 3D Bioprinting. Front. Bioeng. Biotechnol..

[159] Barcena A.J.R., Dhal K., Patel P., Ravi P., Kundu S., Tappa K. (2023). Current Biomedical Applications of 3D-Printed Hydrogels. Gels.

[160] Aguilar E., Herrada-Manchón H. (2024). Editorial for the Special Issue “Hydrogels for 3D Printing”. Gels.

[161] Choonara Y.E., Du Toit L.C., Kumar P., Kondiah P.P.D., Pillay V. (2016). 3D-printing and the effect on medical costs: A new era?. Expert Rev. Pharmacoecon. Amp. Outcomes Res..

[162] Almawash S., Osman S.K., Mustafa G., El Hamd M.A. (2022). Current and Future Prospective of Injectable Hydrogels—Design Challenges and Limitations. Pharmaceuticals.

[163] El Sayed M.M. (2023). Production of Polymer Hydrogel Composites and Their Applications. J. Polym. Environ..

[164] Guo J.L., Kim Y.S., Xie V.Y., Smith B.T., Watson E., Lam J., Pearce H.A., Engel P.S., Mikos A.G. (2019). Modular, tissue-specific, and biodegradable hydrogel cross-linkers for tissue engineering. Sci. Adv..

[165] Richbourg N., Wechsler M.E., Rodriguez-Cruz J.J., Peppas N.A. (2024). Model-based modular hydrogel design. Nat. Rev. Bioeng..

[166] Kim S., Chen J., Cheng T., Gindulyte A., He J., He S., Li Q., Shoemaker B.A., Thiessen P.A., Yu B. (2021). PubChem in 2021: New data content and improved web interfaces. Nucleic. Acids Res..

[167] Bandyopadhyay R., Selvakumar K., Mohamed J.M.M., Ebrahim D. (2024). A Review of Process Validation of Hydrogel Formulation. Int. J. Pharm. Phytopharm. Res..

[168] Mohapatra S., Mirza M.A., Hilles A.R., Zakir F., Gomes A.C., Ansari M.J., Iqbal Z., Mahmood S. (2021). Biomedical Application, Patent Repository, Clinical Trial and Regulatory Updates on Hydrogel: An Extensive Review. Gels.

[169] Bonsel J.M., Itiola A.J., Huberts A.S., Bonsel G.J., Penton H. (2024). The use of patient-reported outcome measures to improve patient-related outcomes—A systematic review. Health Qual. Life Outcomes.

[170] Omidian H., Akhzarmehr A., Chowdhury S.D. (2024). Advancements in Cellulose-Based Superabsorbent Hydrogels: Sustainable Solutions across Industries. Gels.

[171] Kerpel-Fronius S., Becker S., Barrett J., Brun J., Carlesi R., Chan A., Collia L.F., Dubois D.J., Kleist P., Koski G. (2018). The Shared Ethical Responsibility of Medically and Non-medically Qualified Experts in Human Drug Development Teams. Front. Pharmacol..

[172] Hey S.P. (2018). Ethical Challenges in Biomarker-Driven Drug Development. Clin. Pharmacol. Ther..

[173] O’Sullivan L., Aldasoro E., O’Brien Á., Nolan M., McGovern C., Carroll Á. (2022). Ethical values and principles to guide the fair allocation of resources in response to a pandemic: A rapid systematic review. BMC Med. Ethics.

[174] Cheng F., Ma Y., Uzzi B., Loscalzo J. (2020). Importance of scientific collaboration in contemporary drug discovery and development: A detailed network analysis. BMC Biol..

[175] Zhan W., Wang C.-H. (2023). Multiphysics Simulation in Drug Development and Delivery. Pharm. Res..

[176] Lyu X., Hu Y., Shi S., Wang S., Li H., Wang Y., Zhou K. (2023). Hydrogel Bioelectronics for Health Monitoring. Biosensors.

[177] Quazi M.Z., Hwang J., Song Y., Park N. (2023). Hydrogel-Based Biosensors for Effective Therapeutics. Gels.

[178] Zhu Y., Zeng Q., Zhang Q., Li K., Shi X., Liang F., Han D. (2020). Temperature/near-infrared light-responsive conductive hydrogels for controlled drug release and real-time monitoring. Nanoscale.

[179] Campbell K.T., Wysoczynski K., Hadley D.J., Silva E.A. (2020). Computational-Based Design of Hydrogels with Predictable Mesh Properties. ACS Biomater. Sci. Amp. Eng..

[180] Yoon J., Han H., Jang J. (2023). Nanomaterials-incorporated hydrogels for 3D bioprinting technology. Nano Converg..

[181] Lei Y., Santos H.A., Cui W. (2024). Advances and challenges in hydrogel microspheres for biomedical applications. Biomater. Transl..

[182] Auvinen V.-V., Laurén P., Shen B., Isokuortti J., Durandin N., Lajunen T., Linko V., Laaksonen T. (2022). Nanoparticle release from anionic nanocellulose hydrogel matrix. Cellulose.

[183] Chakraborty A., Roy A., Ravi S.P., Paul A. (2021). Exploiting the role of nanoparticles for use in hydrogel-based bioprinting applications: Concept, design, and recent advances. Biomater. Sci..

